# Cortical plasticity in central vision loss: Cortical thickness and neurite structure

**DOI:** 10.1002/hbm.26334

**Published:** 2023-05-17

**Authors:** Matthew K. Defenderfer, Pinar Demirayak, Leland L. Fleming, Dawn K. DeCarlo, Paul Stewart, Kristina M. Visscher

**Affiliations:** ^1^ Civitan International Research Center University of Alabama at Birmingham Birmingham Alabama USA; ^2^ Department of Neurobiology, Heersink School of Medicine University of Alabama at Birmingham Birmingham Alabama USA; ^3^ Department of Ophthalmology, Heersink School of Medicine University of Alabama at Birmingham Birmingham Alabama USA

**Keywords:** central vision loss, cortical thickness, macular degeneration, NODDI

## Abstract

Late‐stage macular degeneration (MD) often causes retinal lesions depriving an individual of central vision, forcing them to learn to use peripheral vision for daily tasks. To compensate, many patients develop a preferred retinal locus (PRL), an area of peripheral vision used more often than equivalent regions of spared vision. Thus, associated portions of cortex experience *increased* use, while portions of cortex associated with the lesion are *deprived* of sensory input. Prior research has not well examined the degree to which structural plasticity depends on the amount of use across the visual field. Cortical thickness, neurite density, and orientation dispersion were measured at portions of cortex associated with the PRL, the retinal lesion, and a control region in participants with MD as well as age‐matched, gender‐matched, and education‐matched controls. MD participants had significantly thinner cortex in both the cortical representation of the PRL (cPRL) and the control region, compared with controls, but no significant differences in thickness, neurite density, or orientation dispersion were found between the cPRL and the control region as functions of disease or onset. This decrease in thickness is driven by a subset of early‐onset participants whose patterns of thickness, neurite density, and neurite orientation dispersion are distinct from matched control participants. These results suggest that people who develop MD earlier in adulthood may undergo more structural plasticity than those who develop it late in life.

## Graphical abstract

Retinal sensitivity is measured in both eyes of patients with central vision loss due to macular degeneration. Instead of using the fovea for daily tasks as most people with healthy vision do, many patients use a “Preferred retinal locus” instead. We ask how the anatomy of the cortical projection of that preferred retinal locus changes with experience.

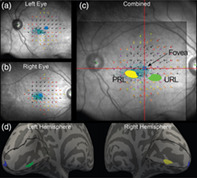

## 1 INTRODUCTION

Macular degeneration (MD) is a retinal disease that, in its late stages, results in loss of central vision but retained peripheral vision, a drastic change in human visual experience. This loss of central vision causes a decrease in the patient's ability to perform visual tasks requiring high visual acuity, such as reading and recognizing faces (Legge et al., 1985). Many patients learn to compensate for the loss of central vision through development of a preferred retinal locus (PRL), a discrete area of usable retina used preferentially for fixation (Crossland et al., 2005). MD has been used as a model for adult cortical plasticity in early visual cortex because of these large‐scale shifts in use of visual space. A number of studies have investigated the effects of input deprivation in the lesion projection zone (LPZ), the area of cortex that typically receives inputs from the scarred portion of the retina, and have found evidence suggesting cortical reorganization that allows processing of peripheral visual signals in deafferented central vision (Baker et al., 2005, 2008; Darian‐Smith & Gilbert, 1994; Dilks et al., 2014); however, the existence, extent, and conditions for this reorganization are disputed (Baseler et al., 2011; Masuda et al., 2008; Smirnakis et al., 2005). Cat and macaque research has shown that axons can grow several millimeters in response to vision loss (Darian‐Smith & Gilbert, 1994); this is thought to result in changes in receptive field size and position after vision loss (Yamahachi et al., 2009). These results imply some level of axonal/dendritic remodeling that has not been fully explored in humans. Neurite orientation dispersion and density imaging (NODDI; Zhang et al., 2012) allows for investigation of changes to neurite structure in living human tissue using specialized diffusion magnetic resonance imaging (MRI) scans.

Cortical thickness is another structural measurement that could change due to vision loss. Previous research has examined differences in cortical thickness across all of primary visual cortex (V1) by comparing MD patients to controls. In particular, Prins et al. (2016) found significantly thinner cortex in the part of V1 that processes central vision (the LPZ), in juvenile‐onset MD (JMD) patients compared with controls, but this difference was not as robust in the patients with age‐related macular degeneration (AMD). Burge et al. (2016) found significantly thicker cortex in a combination of early‐onset and late‐onset MD patients compared with controls at the region in V1 that processes mid‐peripheral vision, near the boundary of the LPZ. Both of these results indicate some form of change in cortical structure due to loss of central vision, but the ROIs used in these studies are too large to say whether these changes in thickness are localized to specific cortical regions of greatly increased use, that is, the projection of the PRL.

The goal of this study was to investigate potential differences in levels of plasticity based on the amount of use of specific areas of the visual field. We test the hypothesis that cortical structure is impacted by (1) increased reliance on information processed at a cortical region of interest (ROI), separately from (2) deprivation of sensory input, or (3) relatively unchanged use. Another goal was to determine how much the age of disease onset influences structural plasticity in participants whose vision loss occurred well after the critical period. Using a microperimetry visual assessment that can map the location of the PRL in the visual field and a technique to accurately transfer the PRL from visual space to cortical space (Defenderfer et al., 2021), we aimed to characterize differences in cortical thickness and neurite structure between healthy participants and controls. Importantly, this approach allows for use of within‐participant control regions that reduce uncertainty from across‐participant variability. We can examine these effects in participant‐specific, retinotopically precise regions defined by increased attentive use (cortical projection of the PRL, cPRL), decreased use (LPZ), and no drastic change in attentive use (cortical projection of unpreferred retinal locus [cURL]), which will inform our understanding of whether these effects reflect use‐dependent plasticity versus deafferentation.

## 2 MATERIALS AND METHODS

### 2.1 Participants

Imaging data were acquired from 34 (20 MD, 14 control) participants. Demographics and visual health details for each MD participant can be seen in Table 1. PRL locations in both eyes as well as notes about vision for each participant with MD are shown in Figure S1. MD participants had been previously diagnosed by an ophthalmologist as having a retinal disease that occluded central, but not all of peripheral, vision. To be enrolled, each MD participant had to have been diagnosed for at least 2 years prior to being enrolled in the study and needed to have bilateral dense scotomas at the center of the fovea. Participants previously diagnosed with MD were divided into groups of early‐onset and late‐onset MD based on whether the age of diagnosis was before or after the age of 30. Most of the “early” onset participants had a form of the disease called Stargardt's macular dystrophy, which tends to present in the teens to early 20s (North et al., 2014). It is important to note that two participants diagnosed with AMD, MDP027 and MDP043, were grouped with the other early‐onset participants. This was because, despite the title of their diagnoses, MDP043 had an age of onset of 18 years, whereas MDP027 had an onset of 21 years, very similar to the onsets of the other early‐onset MD participants (14, 15, 18, and 20 years old). Importantly, even the early‐onset participants had onset well past the “critical period” for vision, which ends during childhood in humans (Levi, 2005). Each control was matched one‐to‐one to an MD participant based on age, gender, and attained education level, but not all MD participants had a corresponding control. Early‐onset MD participants and their controls accounted for 11 total participants (6 MD [3 female, 3 male], 5 controls [3 female, 2 male]), whereas late‐onset MD accounted for 23 participants (14 MD [7 female, 7 male], 9 controls [4 female, 5 male]) . We began with a strategy to have each MD participant age, gender, and education matched to one individual healthy participant. Due to the outbreak of COVID‐19 in early 2020 and considering the safety of the older adults who comprised the large majority of our sample, we were unable to recruit one‐to‐one matches for every MD participant. Although not all MD participants had a healthy matched control, the groups were well matched on average, allowing for careful comparisons between the groups. MD participants did not significantly differ in age from controls in either the early‐onset group (mean ± SD, MD: 47.8 ± 20.8, HC: 43.6 ± 22.2; *t*[8.35] = −0.302, *p* = .770) or the late‐onset group (MD: 70.9 ± 11.4, HC: 69.0 ± 10.9; *t*[17.6] = −0.392, *p* = .700), and the numbers of each gender were the same between control and MD participants. Early‐onset MD participants had significantly worse visual acuity than the late‐onset group (mean ± SD, EO: 1.12 ± 0.21 logMAR, LO: 0.73 ± 0.40 logMAR; *t*[16.6] = −2.83, *p* = .012). Written informed consent was obtained from all participants prior to enrollment in the study.

**TABLE 1:** Macular degeneration participant's demographics and information about use of vision.

Participant	Diagnosis (group)	Sex	Age (y)	Age onset (y)	Dis Dur (y)	Better eye	Acuity (log MAR)	PRL Ecc (°)	PRL size 63% BCEA (°^2^)
MDP004	Age‐related (LO)	F	73	56	17	Right	0.2	4.85	7.2
MDP006	Age‐related (LO)	F	50	46	4	Left	0.9	4.30	6.8
MDP008	Age‐related (LO)	F	70	50	20	Left	0.9	12.9	54.5
MDP014	Age‐related (LO)	M	68	34	34	Left	1.3	10.13	10.1
MDP016	Age‐related (LO)	M	55	42	13	Right	0.2	17.1	9.3
MDP022	Age‐related (LO)	F	83	70	13	Right	0.7	6.98	18.8
MDP047	Age‐related (LO)	M	77	71	6	Right	0.2	5.14	11.4
MDP050	Age‐related (LO)	M	83	73	10	Left	0.8	7.79	12.2
MDP065	Age‐related (LO)	F	61	38	23	Left	0.9	12.56	27.4
MDP117	Age‐related (LO)	M	88	82	6	Left	0.2	5.19	9.7
MDP123	Age‐related (LO)	F	70	66	4	Right	1.0	6.15	5.5
MDP126	Age‐related (LO)	M	83	65	18	Right	0.6	3.33	2.7
MDP142	Age‐related (LO)	M	75	67	8	Right	1.0	13.96	12.7
MDP174	Age‐related (LO)	F	57	47	10	Left	1.3	9.46	84.4
MDP005	Juvenile‐Onset (EO)	M	22	15	7	Right	0.8	10.88	9.7
MDP021	Stargardt's (EO)	M	27	14	13	Left	1.0	9.16	36.3
MDP023	Stargardt's (EO)	F	35	20	16	Right	1.3	8.19	12.0
MDP027	Age‐related (EO)	F	68	21	47	Left	1.0	19.62	22.1
MDP043	Age‐related (EO)	M	65	18	47	Left	1.3	12.83	59.9
MDP122	Juvenile‐Onset (EO)	F	70	19	51	Left	1.3	12.37	16.9

*Note*: Specific diagnosis is given for participants where that information was known along with the onset group they were assigned to (EO, early‐onset; LO, late‐onset). For participants who were not sure of the exact type of juvenile‐onset degeneration, the diagnosis was noted as “juvenile‐onset.” Two participants who reported being diagnosed with age‐related macular degeneration (AMD) also reported the time of diagnosis was before 30 years old. They were ultimately included in the early‐onset group despite their official diagnosis of “AMD” because our hypotheses are about the age of onset, rather than the etiology of the disease. Units are in parentheses following each heading. “Age Onset” is the age of onset as reported by the participant. “Dis Dur” is the duration of disease in years. “Better Eye” is reported by the participant, and is the eye typically with better fixation. “Acuity” is measured with both eyes open. “PRL Ecc” is the eccentricity of the center of the PRL of the better eye (as defined through MAIA). “PRL Size” is the Bivariate Contour Ellipse Area encompassing 65% of all fixations, in units of degrees squared. More information about the MAIA tests for each participant and the resulting cortical regions of interest can be found in Figure S1.

Abbreviation: BCEA, bivariate contour ellipse area.

### 2.2 Scan acquisition

All images were acquired on a 3 T Siemens Prisma scanner using a 64‐channel head coil at our site in Birmingham, Alabama. T1‐weighted (T1w) and T2‐weighted (T2w) scans were acquired for anatomical reference (T1w: MPRAGE, TR/TE = 2400/2.22 ms, 0.8 mm isotropic voxels; T2w: SPACE, TR/TE = 3200/563 ms, 0.8 mm isotropic voxels). Four diffusion‐weighted runs were acquired (*b* = 0/1500/3000 s/mm^2^, TR/TE = 3230/89.20 ms, field of view = 210 mm × 210 mm, multiband acceleration factor = 4, 92 slices, interleaved acquisition, 1.5 mm isotropic voxel size). During the four diffusion MRI runs, q‐space is densely sampled with 185 diffusion‐weighting directions, each acquired twice with opposite phase encoding direction (anterior to posterior (AP), and posterior to anterior (PA); Harms et al., 2018). These 185 diffusion‐weighted directions are split into two sets consisting of 98 and 99 directions, counting the b0 volumes, each taken AP and PA. 98‐direction and 99‐direction scan pairs were processed separately, giving sets of results for 98‐direction and 99‐direction scan pairs. All 4 scans were acquired for every participant except for one late‐onset MD participant where only the 98‐direction pair was acquired. Each 98‐direction scan contained 92 diffusion‐weighted volumes (46 each of *b* = 1500 and 3000 s/mm^2^) in addition to 7 non‐diffusion‐weighted (*b* = 0 s/mm^2^) volumes. For the 99‐direction scans, 93 diffusion‐weighted volumes were collected (47 *b* = 1500 and 46 *b* = 3000 s/mm^2^) in addition to 7 nondiffusion‐weighted volumes.

### 2.3 Data preprocessing

Results included in this manuscript come from preprocessing performed using *fMRIPprep* 1.2.5 (Esteban et al., 2019), which is based on *Nipype* 1.1.6 (Gorgolewski et al., 2011), and described in detail in the following paragraphs.

#### 2.3.1 Anatomical data preprocessing

T1w images were corrected for intensity nonuniformity using N4BiasFieldCorrection (ANTS 2.2.0; Tustison et al., 2010). The T1w image was then skull‐stripped using antsBrainExtraction (ANTs 2.2.0). Brain surfaces were reconstructed using recon‐all, using information from the T2 image to refine placement of the pial surface (FreeSurfer 6.0; Dale et al., 1999), and the brain mask estimated previously was refined with a custom variation of the method to reconcile ANTs‐derived and FreeSurfer‐derived segmentations of the cortical gray matter of Mindboggle (Klein et al., 2017). Spatial normalization to the ICBM 152 Nonlinear Asymmetrical template version 2009c (Fonov et al., 2009) was performed through nonlinear registration with antsRegistration (Avants et al., 2008), using brain‐extracted versions of both T1w volume and template. Brain tissue segmentation of cerebrospinal fluid, white matter and gray matter was performed on the brain‐extracted T1w using fast (Zhang et al., 2001).

Freesurfer‐derived cortical thickness for any given vertex was normalized to the mean hemispheric thickness that vertex belonged to in each individual. This was done in order to account for individual differences in overall mean cortical thickness that could affect cross‐participant comparisons. All models and analyses involving cortical thickness are performed on this normalized thickness as opposed to raw thickness.

#### 2.3.2 Diffusion and NODDI preprocessing

All preprocessing steps were performed identically on 98‐direction and 99‐direction pairs. Raw scans, *b*‐vectors, and *b*‐values files were concatenated across AP–PA pairs before preprocessing to increase the signal‐to‐noise ratio. Standard diffusion preprocessing was performed including correction for motion, eddy currents, and susceptibility artifacts using FSL's *topup* and *eddy* tools (FLS 6.0; Andersson et al., 2003; Smith et al., 2004).

NODDI analysis was performed using the NODDI toolbox (nitrc.org, version 1.01). The full‐brain mask output from FSL's *eddy* was used as the input for NODDI, selecting only gray matter voxels on which to perform NODDI. Previous papers have estimated the intrinsic diffusivity coefficient for optimal calculation of NODDI outcomes in varying tissue types (Fukutomi et al., 2018, 2019; Guerrero et al., 2019); correspondingly, we set the intrinsic diffusivity coefficient for these analyses to 1.1 × 10^−9^ to reflect that the ROIs are in gray matter. This choice of intrinsic diffusivity coefficient is different from the default, set for white matter, and should not impact the group comparisons, since all ROIs from all individuals are in the same tissue type.

The NODDI pipeline resulted in volumes containing intracellular volume fraction (FICVF, a surrogate of neurite density) as well as orientation dispersion index (ODI) for each voxel in the brain mask for each individual, all of which are in native diffusion space after unwarping. These FICVF and ODI volumes were then transformed into the individual's native FreeSurfer space and converted to surfaces using FreeSurfer's *mri_vol2surf*. FICVF and ODI values were then extracted from each vertex within the LPZ, PRL, and URL ROIs from each participant.

### 2.4 Retinal ROI definition

For this study, three different ROIs in visual space were defined: a PRL, a control URL, and a LPZ. Macular integrity assessment (MAIA) microperimetry was performed on each participant, on each eye, if possible (in some cases one eye had vision too poor to perform this assessment). Microperimetry involves simultaneously imaging the retina, and presenting visual stimuli to precise, stabilized locations on the retina. Preferred retinal loci were defined by instructing each participant to fixate a dot and measuring the location on the retina that the participant used for fixation. The center of the PRL was defined as the center of mass of retinal fixation locations over time, and the area of the PRL was calculated as the bivariate contour ellipse area (BCEA) on the retina (Crossland, Sims, et al., 2004) that encompassed 63% (or 95%) of all fixation locations. When possible, microperimetry was performed on both eyes; however, only the PRL from the better functioning eye (as reported by the participant) was used as the PRL. The MAIA assessment outputs two separate BCEA values for each assessment, one that contains 63% of fixations and one that contains 95% of fixations. The BCEA used for the PRL ROI was chosen on a case‐by‐case basis depending on eccentricity from the fovea. In order to partially account for cortical magnification in the visual cortex, the 95% BCEA was chosen for locations further from the fovea to increase the number of cortical vertices in the ROI.

The MAIA assessment also allows measurement of the perceptual sensitivity at 65 locations in the visual field in an individual. Participants were asked to fixate on the center while visual stimuli were presented. Participants were instructed to respond via button press whenever they perceived a light flash. Detection thresholds for these stimuli were calculated for each location on the retina.

In order to align these visual field maps between the eyes, the fovea was manually marked in each MAIA image via estimated distance from the optic disc and vascular landmarks (Rohrschneider, 2004). Images from both eyes were then aligned in Adobe Photoshop so that their foveal locations aligned, making an overlapped MAIA image. This gave a representation of bilateral retinal sensitivity across visual space for an individual, identifying regions of visual space where both eyes had lesions. The URL was then defined as an area of equal eccentricity with the same shape as the PRL in an area of the visual field that still had retinal sensitivity in at least one eye. This area was created by duplicating the PRL and either rotating around or flipping across the fovea using Adobe Photoshop. Each URL location was chosen to maximize the amount of usable vision within the ROI according to the MAIA image.

For certain participants (see Figure S1 for images of the PRL and URL defined for each participant), the BCEA of the PRL partially encroached areas where no visual function existed in either eye, that is, into the overlapping lesion, meaning the participant placed the fixation mark inside a retinal lesion. In these cases, the area with no functional vision in either eye was removed from the overall retinal PRL, creating a nonelliptical PRL shape. In these cases, the URL was defined with an identical shape to the PRL.

PRL eccentricity was measured for each MD participant in both onset groups. Eccentricity was manually calculated in Photoshop as pixel distance from the fovea to the center of the BCEA multiplied by the pixel‐per‐degree conversion factor supplied by the MAIA. According to an independent‐samples *t*‐test, there was no significant difference in PRL eccentricity between the early‐onset and late‐onset groups (mean ± SD, EO: 12.2° ± 4.1°, LO: 8.6° ± 4.2°; *t*[9.88] = 1.80, *p* = .10).

### 2.5 Transfer of retinal ROIs to cortical space

The method for transferring retinal space ROIs to the cortex is described in detail in our recent paper (Defenderfer et al., 2021). In brief, binary PRL and URL ROIs were created in retinal space using Photoshop. A general retinotopic atlas was transferred to each participant's cortical surfaces through the Python library neuropythy (Benson & Winawer, 2018). This atlas assigns visual eccentricity and polar angle to each vertex in a number of visual areas including V1. Each pixel in the PRL and URL images was assigned an eccentricity and polar angle value based on pixel distance from the center of the fovea ROI multiplied by a pixel to visual degree conversion factor obtained from the MAIA microperimeter. For each vertex in V1, the retinotopic coordinates were extracted from the retinotopic atlas, and the pixel with the closest eccentricity and polar angle values was found. If that pixel was located in the PRL, that vertex was added to the cPRL label. The same method was performed for the cURL label as well. For those participants with a PRL or URL that spanned across left and right visual hemifields, those corresponding cortical ROIs would have pieces on both left and right hemispheres. Each cPRL and cURL was then dilated until it had a minimum of 50 vertices. If a given ROI spanned across hemispheres, the larger portion was dilated, whereas the smaller portion remained constant. Any vertices found to be outside of the atlas's V1 boundaries after dilation was removed from the label before counting the final number of vertices in the label. Dilation continued until both portions had combined 50 vertices after removal of non‐V1 vertices. Control participants were assigned a cPRL and cURL label that matched their corresponding MD participant's. Overall, our participants' PRL and URL ROIs had a number of vertices ranging from 55 to 1073 with a mean and standard deviation of 260 ± 207 vertices.

**FIGURE 1:**
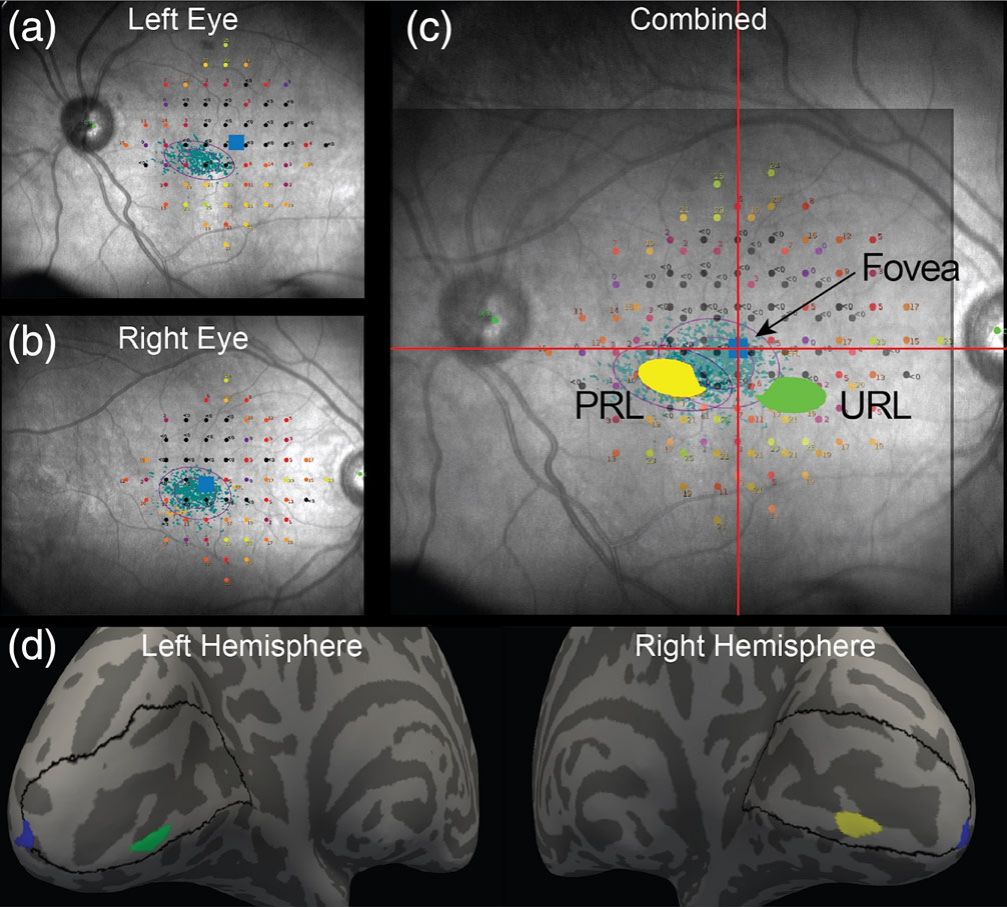
Example macular integrity assessment (MAIA) with marked preferred retinal locus (PRL) and unpreferred retinal locus (URL) converted from retinal space to cortical space. (a) A MAIA microperimetry assessment in an example participant's left eye. The estimated location of the center of fovea is marked with a blue square. Foveal marker size has been increased for readability (same for b,c). Retinal areas with no visual sensitivity are marked with black dots. Visually sensitive areas of the retina are marked with colored dots. Red indicates low visual sensitivity (unhealthy) whereas green indicates high sensitivity (healthy). Numbers are added next to each dot indicating sensitivity level with high numbers describing high sensitivity. Fixation locations during the task are marked by cyan points. The large and small purple circles mark the 95% and 63% bivariate contour ellipse area (BCEA) for fixation, respectively. (b) MAIA assessment in the right eye, same parameters as left eye. Note that for this eye, the participant orients a target to the scotoma (area with black dots), indicating they are unlikely to be able to see the target. (c) MAIA images from both eyes are overlaid and aligned with respect to the fovea. This participant reported their left eye as their better eye so the BCEA from the left eye MAIA was chosen as the PRL (yellow). The PRL was flipped across the vertical meridian (red line), to a spot of equivalent MAIA performance, to create the URL (green). The right eye PRL was ignored, as daily use involves binocular viewing. (d) cortical projection of the PRL regions of interest (ROIs) in yellow, cortical projection of URL in green, and lesion projection zone (LPZ) in blue are shown on the participant's left and right hemispheres (posterior, medial view). The LPZ ROI was defined as the 100 vertices with the lowest assigned eccentricity values in both hemispheres in V1. The border of V1 is marked in black outline.

An LPZ ROI was created for each participant that included the 100 vertices on each hemisphere with the lowest eccentricity values (200 total vertices per participant). These were created for every MD and control participant individually (Figure S1) and illustrated for a representative participant in Figure 1. All cPRL, cURL, and LPZ vertices were subject to the exclusion criteria as described in Section 2.6 for the vertices included in the cortical thickness model. On average, the LPZ ROI subtended 0.386° ± 0.039° (mean ± SD) visual angle across all 34 participants. These general LPZ ROIs represent deafferented tissue in our MD participants, as each of the MD participants had a dense scotoma that included at least the central 0.38° of vision. These ROIs generally match the number of vertices in the PRL and URL ROIs (median ± SD; PRL: 305 ± 246, URL: 184 ± 212, LPZ: 199 ± 5).

### 2.6 Eccentricity ROIs

**FIGURE 2:**
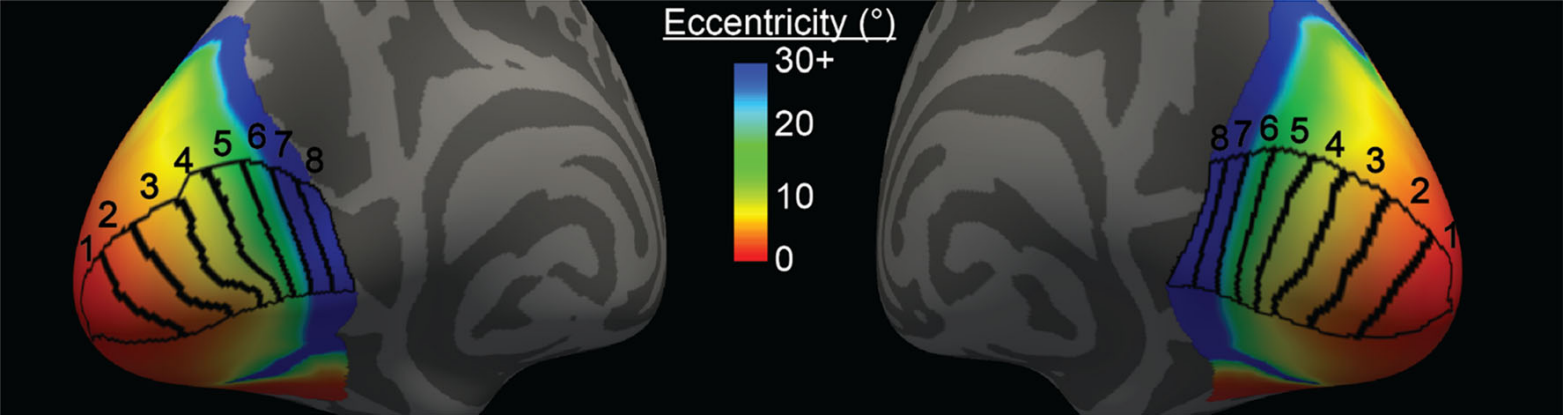
V1 Eccentricity regions of interest (ROIs). Inflated left and right hemispheres of *fsaverage* brain viewed from posterior and medial side. The eight eccentricity ROIs are outlined in black. The eccentricity value of each vertex in the atlas is colored according to the color bar. All vertices with eccentricity >30° are colored dark blue. Eccentricity ROIs were created by gathering *fsaverage* V1 vertices and equally dividing them based on their eccentricity values. Eccentricity ranges are as follows: ROI no. 1: 0°–2.0°, ROI no. 2: 2.0°–4.3°, ROI no. 3: 4.3°–6.8°, ROI no. 4: 6.8°–9.3°, ROI no. 5: 9.3°–15.7°, ROI no. 6: 15.7°–26.6°, ROI no. 7: 26.6°–45.4°, ROI no. 8: 45.4°–90°.

To investigate broad changes in cortical structure across V1 due to vision loss, we created ROIs similar to those seen in Burge et al. (2016) on the surface of *fsaverage* using eccentricity values assigned by the Benson atlas (Benson et al., 2014; Benson & Winawer, 2018). Vertices from both left and right hemisphere V1 were divided into 16 approximately equally sized ROIs (8 per hemisphere) based on their assigned eccentricity in the retinotopic atlas (Figure 2). Eccentricity limits for each set of eight ROIs were the same across hemispheres. Eccentricity ranges were as follows: ROI no. 1: 0°–2.0°, ROI no. 2: 2.0°–4.3°, ROI no. 3: 4.3°–6.8°, ROI no. 4: 6.8°–9.3°, ROI no. 5: 9.3°–15.7°, ROI no. 6: 15.7°–26.6°, ROI no. 7: 26.6°–45.4°, and ROI no. 8: 45.4°–90°. For analyses using these ROIs, whole‐V1 thickness and NODDI metrics for each participant were converted to *fsaverage* space.

### 2.7 Statistical methods

Analyses of Variance (ANOVA) were performed to investigate the effects of ROI location (either PRL vs. URL or the eight eccentricity ROIs), disease state (MD vs. controls), and disease onset (early vs. late) on normalized cortical thickness, neurite orientation dispersion, and neurite density. Although controls do not express “early‐onset” or “late‐onset” phenotypes, we classify them as such because we are interested in how differences between early‐onset and late‐onset MD participants differ compared with controls. If the same pattern of differences is observed between our two control groups as our two MD groups, then these differences are most likely not due to the effects of loss of central vision and ensuing plasticity. For this reason, the effect of disease onset was not interpreted except as an interaction with diagnosis (MD vs. control). This is the case for all reported tests.

Our sample of MD participants in this study had PRLs with varying eccentricities as well as PRL and URL ROI sizes, and these differences could have added noise to mask differences in cortical thickness. To account for this in statistical tests at the PRL and URL, the ANOVA was also run as an analysis of covariance (ANCOVA) including as covariates for each participant's average number of vertices of the cPRL and cURL as well as the average eccentricity those vertices represent retinotopically. The eccentricity value was shared between an MD participant and their matched control (if the matched control existed). Average eccentricity was log transformed to better represent distance across cortex (i.e., a 1° eccentricity change near the occipital pole has a much larger difference in cortical distance than the same change in eccentricity in anterior V1) and was also centered after being log transformed.

Current age was not included in our statistical models because we have included age‐matched controls for each of the early‐onset and late‐onset groups. Although age does have an effect on cortical thickness (Salat et al., 2004), any difference in thickness due to age between the early‐onset and late‐onset MD groups would be similar for the early‐onset and late‐onset control groups. Also, all cortical thickness estimates were normalized to the average hemispheric thickness, which should account for general effects of aging on cortical thickness.

## 3 RESULTS

### 3.1 Normalized cortical thickness

#### 3.1.1 Cortical thickness at retinally specific ROIs

**FIGURE 3:**
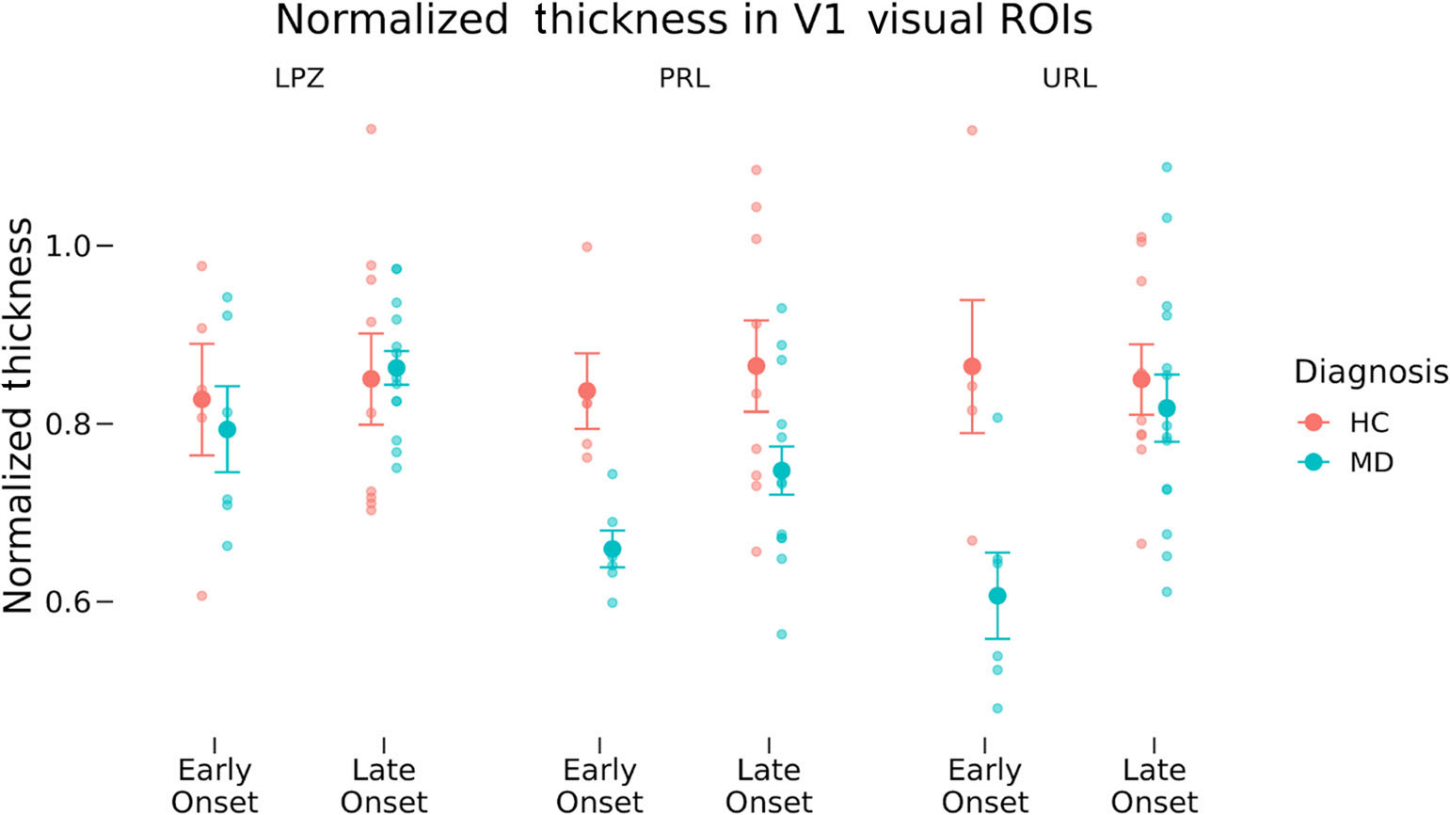
Normalized thickness across region of interest (ROI), diagnosis, and onset groups. Normalized thickness is shown at the three cortical locations: lesion projection zone (LPZ), cortical projection of the preferred retinal locus (cPRL), and cortical projection of unpreferred retinal locus (cURL) as a function of both onset (early vs. late) and diagnosis (macular degeneration [MD] vs. healthy control [HC]). Error bars represent standard error of the mean. Equivalent data for non‐normalized thicknesses are shown in Figure S2.

Normalized thickness values for each participant group in the LPZ, the cPRL, and the cURL are shown in Figure 3. A three‐way mixed ANOVA of normalized cortical thickness as a function of diagnosis (MD or control), MD onset (early or late), and ROI location (cPRL and cURL) was used to test the hypothesis that the PRL, which is used more in daily life than the cURL, will be thicker in the participants with central vision loss but not the controls. We also performed an ANCOVA including the average eccentricity and the average number of vertices in the PRL and URL ROIs. Hypotheses specifically relating to the LPZ were tested separately.

At the cPRL and cURL, we found a significant main effect of MD diagnosis on normalized thickness (*F*[1, 30] = 16.23, *p* = .0004). The two‐way interaction of diagnosis and location was nonsignificant (*F*[1, 30] = 0.002, *p* = .967), and the two‐way interaction of diagnosis and onset (our hypothesized interaction if age of onset impacts thickness) was nonsignificant but trending (*F*[1, 30] = 3.88, *p* = .058). The three‐way interaction between MD diagnosis, onset, and ROI location was non‐significant (*F*[1, 30] = 2.15, *p* = .153). Including average eccentricity and vertex count of the PRL ROI as covariates similarly resulted in a significant main effect of diagnosis (*F*[1, 28] = 16.56, *p* = 0.0003) as well as a significant interaction of diagnosis and onset (*F*[1, 28] = 4.61, *p* = .041), our hypothesized interaction if age of onset impacts thickness. The two‐way interaction of diagnosis and location (*F*[1, 28] = 0.01, *p* = .905), the three‐way interaction of diagnosis, onset, and location (*F*[1, 28] = 2.27, *p* = .143), and the covariates ROI size (*F*[1, 28] = .95, *p* = .338) and eccentricity (*F*[1, 28] = 0.13, *p* = .726) were all nonsignificant.

An interaction of diagnosis and onset indicates that a difference in normalized thickness between early‐onset and late‐onset MD participants varies compared with the difference between their respective controls. To improve the interpretability of this effect, we controlled for visual acuity differences in early‐onset (EO) versus late‐onset (LO) MD participant groups by performing a follow‐up ANCOVA. This ANCOVA examined normalized thickness as a function of ROI location (cPRL and cURL) and onset (early and late) only within the MD group, adding age and visual acuity as covariates along with ROI eccentricity and size (as above). This showed a significant interaction of onset and location on normalized thickness in our MD groups (*F*[1, 14] = 6.90, *p* = .020) and showed no significant effect of either acuity (*p* = .919) or age (*p* = .796). This indicates that any difference between early‐onset and late‐onset MD participants is not due solely to a difference in visual acuity between the groups. The significant interaction of onset by location also indicates that experience using the PRL more than the URL impacts the early and late‐onset MD participants differently. The right side of Figure 3 shows thickness for the PRL and URL ROIs. The image illustrates that this is driven by thinning in the URL for early‐onset participants that is greater than for late onset, whereas for the PRL, the groups have more similar, less profound thinning.

The same tests were performed using raw cortical thickness values (see Figure S2) in order to control for the possibility that normalization might influence the results. Results showed a significant interaction of diagnosis and onset at the PRL and URL (*F*[1, 30] = 6.32, *p* = .018) supporting the effects observed using normalized thicknesses.

LPZ thicknesses showed no significant differences as a function of either diagnosis (*F*[1, 30] = 0.061, *p* = .293) or interaction of onset and diagnosis (*F*[1, 30] = 0.285, *p* = .597).

#### 3.1.2 Cortical thickness at eccentricity ROIs

**FIGURE 4:**
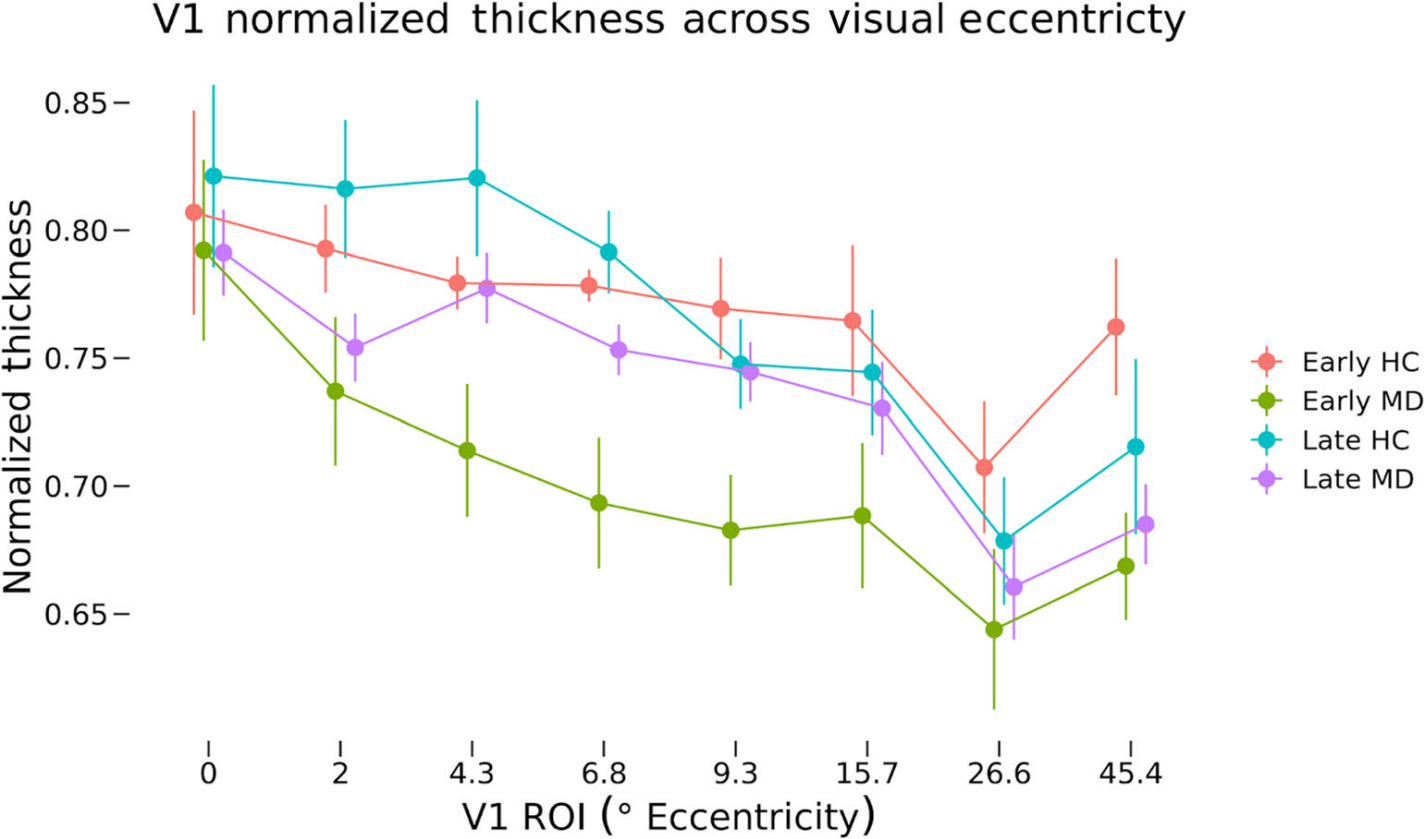
Normalized thickness across eccentricity regions of interest (ROIs). Mean normalized thickness for each macular degeneration (MD) and control group at each eccentricity ROI are shown. Error bars represent ±1 SEM. Values shown on the *x*‐axis correspond to the minimum eccentricity for that ROI. For example, ROI number two has an eccentricity range of 2.0–4.3, so the second label is 2. A three‐way ANOVA of normalized thickness as a function of diagnosis, onset, and ROI was performed. Main effects of diagnosis (*F*[1, 30] = 10.43, *p* = .003) as well as ROI (*F*[3.35, 100.52] = 16.37, *p* = 2.14 × 10^−9^) were significant. No two‐way or three‐way interactions were significant. HC, healthy control.

For each participant, normalized thicknesses were converted to *fsaverage* space and averaged within each eccentricity ROI (Figure 4). We performed a three‐way ANOVA of normalized thickness with between‐participant factors of diagnosis (healthy control [HC] or MD) and MD onset (early or late) as well as the within‐participant factor of ROI. These data violated Mauchley's test for sphericity (*W* = 0.053, *p* = 3.53 × 10^−7^), and so degrees of freedom were adjusted using the Hyuhn‐Feldt epsilon value (*ε*_HF_ = 0.56) for effects of ROI location. We found significant main effects of diagnosis (*F*[1, 30] = 10.25, *p* = .003) as well as ROI (*F*[3.91, 117.2] = 16.37, *p* = 1.59 × 10^−10^). The two‐way interactions of diagnosis and onset (*F*[1, 30] = 1.54, *p* = .224) and diagnosis and ROI (*F*[3.91, 117.2] = 0.43, *p* = .780) were both nonsignificant. The three‐way interaction of diagnosis, onset, and ROI was also nonsignificant (*F*[3.91, 117.2] = 0.72, *p* = .575). This indicates that over the entirety of V1, cortex is thinner across all MD participants compared with controls, but there is not a statistically significant difference in patterns of cortical thickness as a function of age of onset. Current age was not included in the model (see Section 2.7) because we have included age‐matched controls for each of the early‐onset and late‐onset groups.

The same test was performed using raw thickness values (see Figure S3) finding significant main effects of diagnosis (*F*[1, 30] = 10.22, *p* = 003) and ROI (*F*[7, 210] = 16.47, *p* = 3.09 × 10^−17^). The two‐way interaction of diagnosis and onset (*F*[1, 30] = 3.19, *p* = .084) as well as the three‐way interaction of diagnosis, onset, and ROI (*F*[7, 210] = 0.66, *p* = .705) were not significant.

### 3.2 Neurite orientation dispersion and density imaging

#### 3.2.1 NODDI at retinally specific ROIs

**FIGURE 5:**
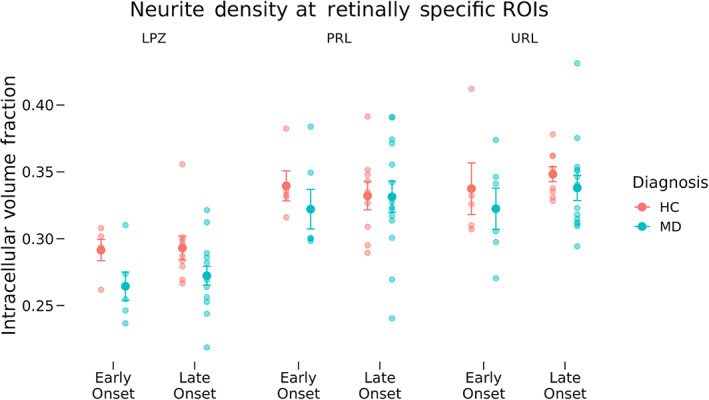
Neurite density across region of interest (ROI), diagnosis, and onset groups. Neurite density as measured by neurite orientation dispersion and density imaging is shown for the lesion projection zone (LPZ), cortical projection of the preferred retinal locus (cPRL), and cortical projection of unpreferred retinal locus (cURL) as a function of diagnosis and disease onset. Error bars represent standard error of the mean. Effects at the LPZ were tested independently from the PRL and URL. Tests at the cPRL and cURL showed no significant differences in neurite density as a function of ROI location, diagnosis or disease onset or any interactions. Significant differences in neurite density were found at the LPZ as a function of diagnosis (*F*[1, 30] = 6.490, *p* = .016), but there was not a significant interaction of diagnosis and disease onset (*F*[1, 30] = 0.112, *p* = .740). HC, healthy control; MD, macular degeneration.

**FIGURE 6:**
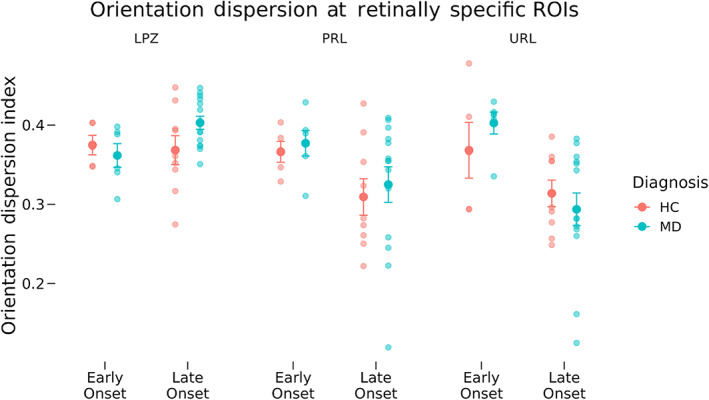
Orientation dispersion across region of interest (ROI), diagnosis, and onset groups. Orientation dispersion as measured by neurite orientation dispersion and density imaging is shown for the lesion projection zone (LPZ), cortical projection of the preferred retinal locus (cPRL), and cortical projection of unpreferred retinal locus (cURL) as a function of diagnosis and disease onset. Error bars represent standard error of the mean. Small values of orientation dispersion mean neurites lay along similar directions to each other. Effects at the LPZ were tested independently from the cPRL and cURL. No significant differences in orientation dispersion were found at the LPZ, cPRL, or cURL. HC, healthy control; MD, macular degeneration.

Neurite density (Figure 5) and orientation dispersion (Figure 6) for each participant group in the LPZ, the cPRL, and the cURL are shown. A three‐way mixed ANOVA as a function of diagnosis, MD onset, and ROI location was used to test the hypothesis that the cPRL will have higher neurite density in the MD participants but not the controls compared with the cURL (Figure 5). At the cPRL and cURL, the main effect of diagnosis was nonsignificant (*F*[1, 30] = 0.852, *p* = .363). The two‐way interaction of Diagnosis and Location was nonsignificant (*F*[1, 30] = 0.094, *p* = .761) and the two‐way interaction of diagnosis and onset was nonsignificant (*F*[1, 30] = 0.208, *p* = .652). The three‐way interaction between diagnosis, onset, and ROI location was also nonsignificant (*F*[1, 30] = 0.271, *p* = .606). In the same fashion as for the normalized thickness analysis, the eccentricity and size of the cPRL and cURL were added as covariates in an ANCOVA and did not change the results in terms of statistical significance from the main ANOVA. Current age was not included in statistical models (see Section 2.7) because we have included age‐matched controls for each of the early‐onset and late‐onset groups.

A similar ANOVA was performed for ODI (Figure 6). At the cPRL and cURL, the main effect of diagnosis was nonsignificant (*F*[1, 30] = 0.203, *p* = .655). The two‐way interaction of diagnosis and location was nonsignificant (*F*[1, 30] = 0.084, *p* = .774) and the two‐way interaction of diagnosis and onset was nonsignificant (*F*[1, 30] = 0.305, *p* = .584). The three‐way interaction between diagnosis, onset, and ROI location was also non‐significant (*F*[1, 30] = 1.983, *p* = .169). The ANCOVA including the effects of cPRL and cURL eccentricity and size did not have different results in terms of statistical significance from the main ANOVA.

Two‐way ANOVAs were performed at the LPZ testing for differences in neurite density as well as ODI as a function of diagnosis and disease onset. *F*or ODI, the main effect of diagnosis (*F*[1, 30] = 0.545, *p* = .466) and the interaction of diagnosis and onset (*F*[1, 30] = 2.689, *p* = .111) were both nonsignificant. However, tests of differences in neurite density at the LPZ found significantly lower density at the LPZ in MD participants compared with HCs, regardless of MD onset (*F*[1, 30] = 6.489, *p* = .016), but the interaction between diagnosis and onset was nonsignificant (*F*[1, 30] = 0.112, *p* = .740). The ANCOVA including the effect of cPRL and cURL eccentricity did not have different results in terms of statistical significance from the main ANOVA.

#### 3.2.2 NODDI at eccentricity ROIs

**FIGURE 7:**
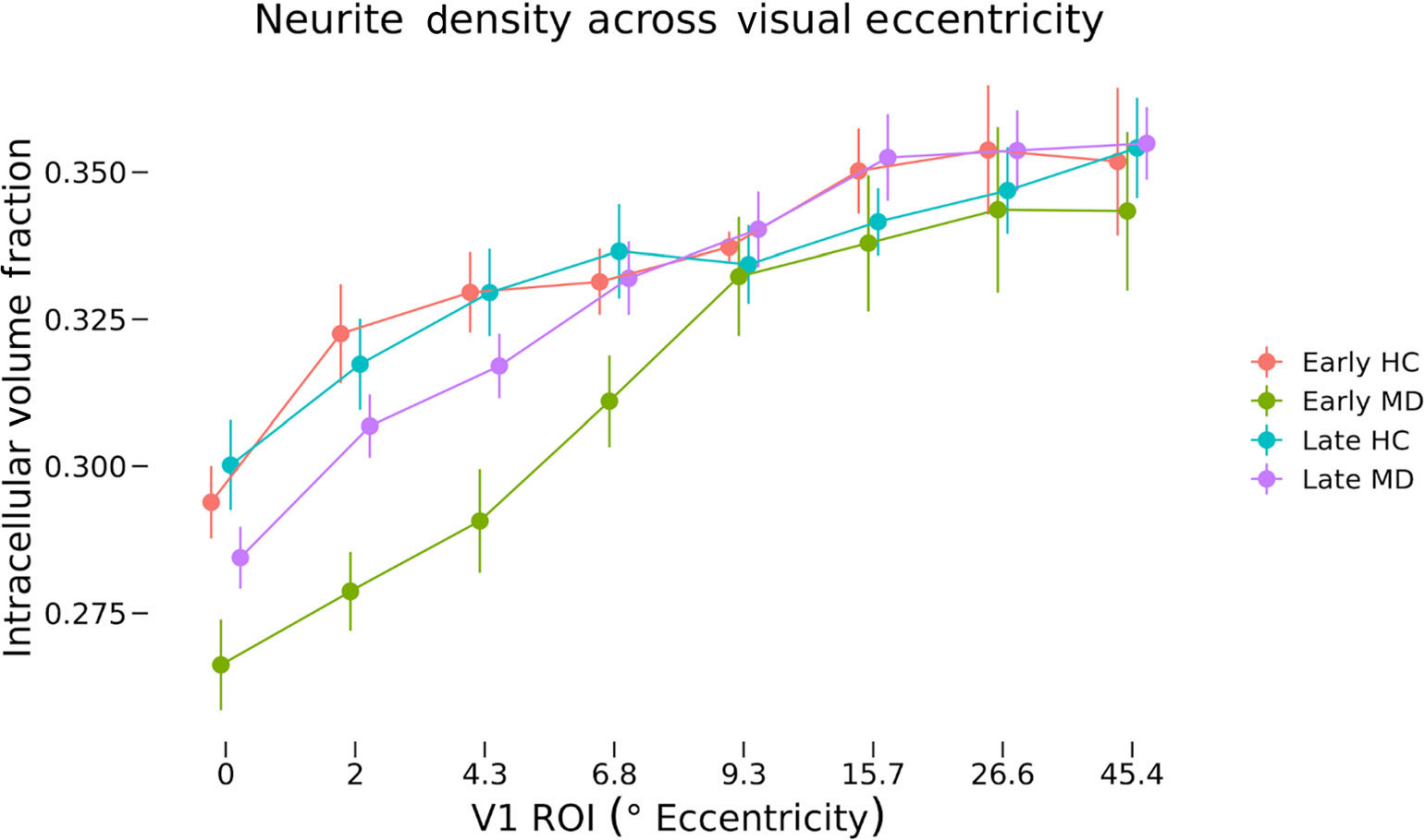
Neurite density across eccentricity regions of interest (ROIs). Mean neurite density for each macular degeneration and control group at each eccentricity ROI are shown. Error bars represent SEM. Values shown on the *x*‐axis correspond to the minimum eccentricity for that ROI. For example, ROI number two has an eccentricity range of 2.0–4.3, so the second label is 2. A three‐way ANOVA of neurite density as a function of diagnosis, onset, and ROI was performed. The main effect of ROI (*F*[4.2126] = 126.85, *p* = 3.53 × 10^−44^) was significant as well as the two‐way interaction of diagnosis and ROI was also significant (*F*[4.2126] = 8.283, *p* = 3.35 × 10^−6^). HC, healthy control.

**FIGURE 8:**
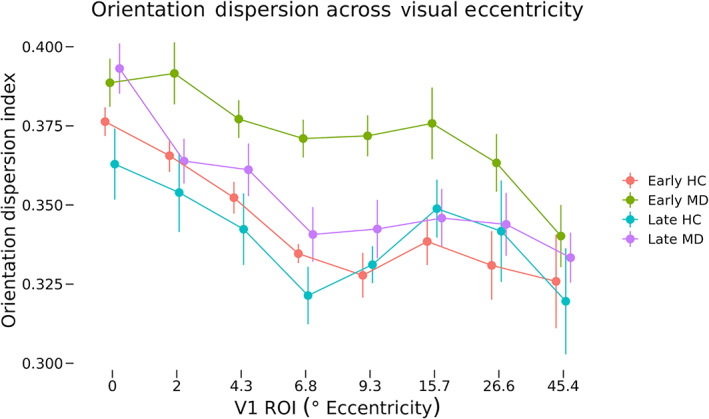
Orientation dispersion across eccentricity regions of interest (ROIs). Mean orientation dispersion for each macular degeneration (MD) and control group at each eccentricity ROI are shown. Error bars represent SEM. Values shown on the *x*‐axis correspond to the minimum eccentricity for that ROI. For example, ROI number two has an eccentricity range of 2.0–4.3, so the second label is 2. A three‐way ANOVA of orientation dispersion as a function of diagnosis, onset, and ROI was performed. Main effects of diagnosis (*F*[1, 30] = 6.73, *p* = .014) as well as ROI (*F*[2.89, 86.73] = 15.04, *p* = 8.50 × 10^−8^) were significant. No two‐way or three‐way interactions were significant. HC, healthy control.

For each participant, neurite density and ODI results were converted to *fsaverage* space and averaged within each eccentricity ROI (Figures 6 and 8, respectively). We performed a three‐way ANOVA of neurite density (Figure 7) with between‐participant factors of diagnosis and MD onset as well as the within‐participant factor of ROI. These data violated Mauchley's test for sphericity (*W* = 0.02, *p* = 6.20 × 10^−11^), and so degrees of freedom were adjusted using the Hyuhn–Feldt epsilon value (*ε*_HF_ = 0.6) for effects of ROI location. The main effect of diagnosis (*F*[1, 30] = 2.38, *p* = .133) was nonsignificant, but the main effect of ROI (*F*[4.2, 126] = 126.85, *p* = 3.53 × 10^−44^) was highly significant. The two‐way interaction of diagnosis and onset (*F*[1, 30] = 1.51, *p* = .229) was nonsignificant, but the interaction of diagnosis and ROI (*F*[4.2126] = 8.28, *p* = 3.85 × 10^−6^) was highly significant. The three‐way interaction of diagnosis, onset, and ROI was nonsignificant (*F*[4.2126] = 1.07, *p* = .374). To further investigate the significant interaction of diagnosis and ROI, post‐hoc tests were performed with independent samples *t*‐tests at each ROI testing differences in neurite density between all controls and all MD participants. Significant differences were found at ROI representing 0°–2° eccentricity (*t*[29] = 2.69, 0.011), ROI representing 2°–4.3° eccentricity (*t*[29.4] = 2.73, *p* = .011), and ROI representing 4.3°–6.8° eccentricity (*t*[31.2] = 2.75, *p* = .010), all of which passed FDR correction for multiple comparisons. No significant differences were found in ROIs 4–8.

A similar three‐way ANOVA was performed for ODI values across the same groups (Figure 8). These data violated Mauchley's test for sphericity (*W* = 0.01, *p* = 2.82 × 10^−14^), and so degrees of freedom were adjusted using the Hyuhn–Feldt epsilon value (*ε*_HF_ = 0.413) for effects of ROI location. The main effects of diagnosis (*F*[1, 30] = 6.73, *p* = .014) as well as ROI (*F*[2.89, 86.73] = 15.04, *p* = 8.50 × 10^−8^) were significant. The two‐way interactions of diagnosis and onset (*F*[1, 30] = 0.97, *p* = .333) as well as diagnosis and ROI (*F*[2.89, 86.73] = 0.36, *p* = .924) were nonsignificant. The three‐way interaction of diagnosis, onset, and ROI was nonsignificant (*F*[2.89, 86.73] = 1.31, *p* = .276).

## 4 DISCUSSION

Using MD as a model of central vision loss allows us to test hypotheses about how cortical structure is impacted by (1) increased reliance on information processed at a cortical ROI, separately from (2) deprivation of sensory input, or (3) relatively unchanged use. We examined whether changes in structure after the onset of MD were most evident in (1) the visual region of highest use (cPRL) when compared with (2) a region that has retained no bottom‐up retinal input (LPZ), as well as (3) a peripheral control region where use of vision was relatively unchanged (cURL). We compared cortical regions with disparate visual use within the same individual to determine how the amount of use impacts structure. This design allowed us to look for experience‐driven plasticity in fine‐grained detail across retinotopically defined regions and to measure effects due to loss of vision as well as an increase in the use of vision. This is important because a change in cortical structure may be dependent on adaptive increased use of spared sensation, and not just sensory loss. Additionally, participants were divided into early‐onset and late‐onset MD groups to explore possible differences in the expression of structural plasticity based on the age of onset of degeneration. Results from the discrete, finely detailed retinotopic regions where differential use was documented were also supplemented by analyses covering large divisions of V1 to better describe how MD affects the primary visual cortex as a whole. Overall, our results show that both cortical thickness and cortical microstructure differ across V1, and differ between groups in ways that are consistent with the idea that there is structural change associated with central vision loss in adulthood. However, this change is limited and, based on our data, seems to depend on vision loss, but not increased use of portions of vision.

### 4.1 Cortical thickness

The representations of peripheral vision in both cPRL and cURL were thinner in MD participants than in controls. The early‐onset MD group showed more thinning than the late‐onset group. Comparisons of normalized thickness values, correcting for whole‐brain atrophy at a participant level, showed a significant interaction between diagnosis and onset group when including ROI eccentricity and size as covariates (Figure 4). Tests using raw thickness values (which does not correct for whole brain atrophy; Figure S2) showed a significant interaction of diagnosis and onset, supporting this result. These results suggest that central vision loss results in thinning of visual cortex associated with peripheral representations, and this effect is greater in those who experience vision loss earlier in adulthood, suggesting that the capacity for neural plasticity (in this case, cortical thinning) is stronger in younger adulthood. While these participants did have spared vision within the PRL and URL, their vision was not as good as HCs. Additionally, due to their central vision loss, MD participants may be more likely to avoid vision‐heavy tasks in their daily life. Thus, the cortical thinning in representations of peripheral vision in MD is consistent with an overall use‐dependent hypothesis for cortical thickness.

Contrary to our initial hypothesis that removal of retinal input would cause a decrease in thickness at the representation of central vision (LPZ), no difference in thickness was found between MD groups and their controls. Interestingly, this seems to be the case only for the representation of the very center of the fovea (Figure 4). When testing normalized thickness across V1, we found that MD participants had significantly thinner cortex than their controls overall. This seems to be driven by the thinner cortex for the early‐onset participants' LPZs, outside of the representation of the central‐most fovea.

The critical test of the hypothesis that increased use of peripheral vision (in the PRL) resulted in thickening of the cortex, was to compare the ROI corresponding to peripheral vision: the cPRL and cURL. While no statistical tests showed significant interactions of ROI (cPRL vs. the cURL) by diagnosis (controls vs. MD), the pattern of data shows that in the participants with early‐onset MD, the URL is more thinned (relative to control) than the PRL. In fact, an ANCOVA examining just the patients with MD showed a significant interaction of age of onset by ROI (cPRL vs. cURL). Participants who had vision loss earlier in adulthood had thin cortex in the URL, consistent with atrophy from disuse, whereas the same participants showed less thinning in the cPRL, consistent with less atrophy from disuse. This was not the pattern for the late‐onset participants, who generally had higher normalized cortical thickness, consistent with less cortical change from their baseline. This suggests the possibility that experience using the PRL influences cortical thickness by counteracting some of the cortical thinning effects of living with MD. Future work with larger numbers of early‐onset MD participants will be needed to more strongly test this hypothesis. The current data put two constraints on the possible effects expected from a larger‐scale study, first suggesting that the effect is likely much stronger in people who have altered vision experience earlier in adulthood. Second, the data suggest that if increased use of a specific piece of cortex (the cPRL) results in cortical thickening, it does so in the context of other processes associated with living with central vision loss that promotes more general visual cortical thinning.

These results are consistent with Prins et al. (2016), who found thinning in a posterior V1 ROI which spanned from the occipital pole to approximately the middle of V1, a region that would represent central vision to ~10° visual eccentricity according to retinotopic atlases (Benson et al., 2012, 2014). They found significantly thinner cortex in their juvenile MD patients in this region, which spans the LPZ used here, as well as the cortical projections of the PRL and the URL. In light of our current results, we may reinterpret Prins's findings to imply not that there is atrophy in the LPZ, but instead atrophy in a wide swath of the visual cortex as a whole. In fact, in the most polar portions of the LPZ, representing vision near the fovea, we did not find thinning in either group. This is consistent with data from Burge et al. (2016), who showed that in the most polar portion of V1, there was no appreciable thickness difference between MD participants versus controls. That group of participants was primarily AMD, and so would have fallen in the “late‐onset” group we studied here. A recent paper (Plank et al., 2021) examined the cortical thickness of cortical projections of a PRL and a control peripheral region similar to our URL. While they found that the cortical projection of the URL was thinner than that of the PRL, this was true in both patients and controls, similar to the finding here. Thus, the data we observed here are consistent with the previous literature, despite the fact that they call into question the interpretations of that literature in some cases.

Burge et al. (2016) also found an overall increase in thickness in the cortical representation near the PRL of patients with MD, a finding that is, at face value, inconsistent with the results observed here. Their methods for defining ROI were the precursor for the eccentricity‐based ROI analysis seen here in Figure 4. Representations of more central vision are thicker than peripheral regions in all groups, consistent with Burge et al. (2016), but a key difference between their results and those seen here is that the increase in thickness in peripherally representing regions around the location of the cPRL is absent in our data. While the data presented here do not invalidate previous results, it is illustrative of sampling effects and the range of individual variability in adaptation to central vision loss. The Burge dataset only included 10 MD participants, which is often considered a moderate sample size for this type of experiment in such well‐characterized patient samples. However, 10 participants (and even the 20 MD participants presented here) may not be large enough to detect reliable effects that apply consistently to the entire population. In order to understand cortical plasticity in the context of central vision loss, it is critical that these results receive further study with larger samples, allowing power to detect differences based on early‐onset versus late‐onset MD.

We did not find cortical thinning associated with the most central part of the LPZ, though all participants had vision loss in that area. While this result was contrary to our initial hypothesis of deprivation‐driven cortical thinning, there was a trend toward cortical thinning in the MD participants just outside the projection of the fovea. This was consistent with a result from Burge et al. (2016), who found no difference between controls and MD subjects when examining the gyrus of the central‐most representation in V1. Further work should identify whether there is something special about the representation of the fovea, which saves it from thinning with deprivation, or whether there are between‐participant differences in experience or behavior, which can account for differences in this effect.

Interestingly, the across‐eccentricity pattern for the controls for late‐onset and early‐onset participants is somewhat different: The slope of change in thickness by eccentricity is greater for the late‐onset participants than for the early‐onset. This is consistent with previous work showing that healthy older adults have a more steep change in cortical thickness with eccentricity than healthy younger adults (Griffis et al., 2016). Our data, coupled with the Prins, Burge, and Plank datasets, suggest that there is great variability in cortical thickness changes following vision loss. Some of this variability may come from differences in time of onset, as suggested by the data in Figures 3 and 4 where there are numerically larger differences between MD and controls in early‐onset participants as well as differences across a broader area of V1 (Figure 4).

In general, our data show an interesting pattern of differences in cortical thickness as a function of eccentricity (thicker cortex in central regions), MD (thinner cortex in patients with vision loss than controls), age (older controls show different slopes of thickness across eccentricity than younger controls), and age of onset (early‐onset MD participants have thinner cortex than the late‐onset MD participants). Taken together with the existing literature, this shows evidence toward the idea that patients with central vision loss exhibit thinning of V1 overall, with sparing of the representations of the center of the fovea.

### 4.2 Neurite orientation dispersion and density imaging

Mirroring analyses of cortical thickness, neurite density, and orientation dispersion were analyzed both at fine‐grain retinotopically defined ROIs corresponding to the cPRL and cURL, as well as in broad regions covering the entirety of V1 to provide additional context to the results. We hypothesized that neurites at the PRL of early‐onset MD participants would restructure and grow to connect to LPZ neurons that lost visual input. This would result in higher neurite density at the cPRL but not the cURL. This would also predict a lower ODI at the cPRL but not the cURL, since many new neurites would be oriented in similar directions toward the LPZ. Analyses of NODDI outputs at the cPRL and cURL found no significant differences in neurite density as a function of either disease or onset (Figures 5 and 6). Patterns of neurite density in the eccentricity ROIs support this finding (Figures 7 and 8). No significant differences were found among the groups in ROIs associated with 6.8° eccentricity and above (the last five ROIs in Figures 7 and 8). These regions are most associated with healthy vision in our participants. Prior research has found evidence of changes in neurite structure in response to loss of vision (Darian‐Smith & Gilbert, 1994; Yamahachi et al., 2009), but these changes were localized to regions just surrounding the LPZ, and were measured in individual cells. Other work has used MRI methods to find evidence of microstructural alterations with vision loss in several brain areas, but not specifically associated with increased and decreased use associated with the PRL and LPZ (Beer et al., 2020).

Neurites reorganizing and growing towards the LPZ to form horizontal connections and recruit deafferented neurons should decrease the orientation dispersion of these neurites, but it is not a given that this effect would be spread evenly across the entire cortical representation of the PRL. The cortical representations of the PRL and URL varied widely in size across participants depending on eccentricity and BCEA (58 vertices to 865 vertices, mean = 272 vertices, SD = 200 vertices). While neurites have the ability to grow over relatively long distances postlesion (e.g., 8 mm in [Yamahachi et al., 2009]), it is reasonable to expect this phenomenon to decrease the further away from the LPZ a specific neuron is. Further investigation is needed specifically in small regions associated with the PRL and URL abutting the LPZ to rule out the hypothesis that neurite orientation and dispersion are modified in MD.

Neurite density at the LPZ was sharply decreased in participants with central vision loss compared with the controls (Figure 5). In contrast to the lack of difference in cortical thickness at the LPZ ROI, this decrease in neurite density was significant in ROIs associated with eccentricities 4.3° and below (first three ROIs in Figure 7). Thus, whatever is keeping cortical thickness the same after deafferentation of foveal vision, it is not the density of neurites. There is evidence for deafferentation‐induced apoptosis of cortical cells, which could contribute to this decrease in neurite density (Capurso et al., 1997; Ginsberg & Martin, 2002; Isono et al., 1997).

When investigating differences in ODI at the retinotopically specific ROIs, no significant difference was found at the cPRL, cURL, or LPZ as a function of onset or diagnosis. However, in the eccentricity ROIs, a significant difference in ODI was found as a function of diagnosis regardless of onset or ROI. Values in Figure 8 indicate this effect is largely driven by higher ODI values across ROIs representing 2°–15.7° in the early‐onset MD group compared with the other groups. Further research is needed to understand why ODI would be higher in the MD participants than in controls.

Overall, these NODDI data provide no evidence for the hypothesis that levels of neurite plasticity are influenced by the amount of visual use in healthy peripheral vision. However, there are differences in density and orientation dispersion as a function of diagnosis, and these differences seem to be more prominent in the early‐onset MD group in deafferented regions. This does suggest that structural plasticity depends to some extent on when MD developed in an individual.

### 4.3 Limitations

While this study allows a careful quantification of the effects of plasticity in a human model, it has some limitations to keep in mind while interpreting these data. This study assumes that only a single PRL is used for each MD participant and that the chosen URL is actually used less in daily life than the PRL. There is ample evidence to suggest that some people develop multiple PRLs that are used for different tasks during daily life (Crossland et al., 2005, 2011; Déruaz et al., 2002; Lei & Schuchard, 1997). If the PRL measured during the microperimetry assessment is not the PRL used for a majority of daily visual tasks, our use of the PRL to assess use‐dependent plasticity may be unwarranted. Likewise, if the chosen URL falls into a secondary PRL but is not identified during microperimetry, it would be reasonable to see very little difference between the cPRL and cURL regions. That said, the PRL as measured with microperimetry is a reliable metric that has been clinically useful in many contexts (Crossland, Culham, & Rubin, 2004), and the accuracy of the measurement has been documented via test–retest of the projection of the same retinal point (Markowitz & Reyes, 2013).

The design was limited by sample size, especially for early‐onset participants. While 6.5% of the US population aged 40 or older is estimated to have some form of AMD (Klein et al., 2011), JMD is much less common. For instance, Stargardt's macular dystrophy, the most common form of JMD, only has an estimated prevalence of 1 in 10,000 (Blacharski, 1988). Having a much smaller JMD population to recruit from decreased our sample size and statistical power. This affected comparisons of cortical thickness, neurite density, and orientation dispersion where there appears to be a trend that our early‐onset participants had more distinct patterns across cortex compared with their controls than the late‐onset participants. Those interactions did not reach statistical significance but suggest that further investigation with a larger early‐onset sample size is necessary. There were also some differences in degree of impairment between the groups, where the early‐onset group had more impaired vision on average.

As with all MRI studies, measurements are affected by proximity to the gradient coils during acquisition. Gradient coil field strength decreases as one moves from the edge to the center of the bore; this can affect contrast in the images. While this could lead to discrepancies in cortical thickness during surface reconstruction, it should do so in all participants and should not affect controls differently from MD; the same is true with early‐onset versus late‐onset groups.

We found that early‐onset MD participants showed thinner cortex overall than their matched controls, and they also showed higher overall ODI and lower neurite density. As stated earlier, this is potentially interesting as it suggests that early‐onset MD may lead to a remodeling of cortex that decreases neurite density while also decreasing cortical thickness, consistent with a loss of cells. This interpretation is tempered by the fact that partial volume effects where voxels on thinner cortex included slightly more CSF would lead to smaller ODI and neurite density values. On the other hand, partial volume effects where voxels on thinner cortex included more white matter would likely have the opposite effect suggesting that partial volume effects should not bias the relationship of cortical thickness to ODI and neurite density. However, future work, perhaps with smaller voxel sizes and therefore less influence of partial volume effects, is needed to entirely disambiguate cortical thickness versus ODI and neurite density values.

## 5 CONCLUSION

This study is novel because the design allowed examination of plasticity in a human model, where we can look independently at the effects of deprivation (LPZ) and increased use (cPRL), relative to a control region that is neither deprived nor strongly attended in daily life (cURL). This capability is due to the extremely well‐characterized central vision loss of these participants. Overall, our data provide evidence that MD influences cortical structure. While there is a great deal of participant‐to‐participant variance, we saw that overall cortical thickness decreased with experience of vision loss in this population. Consistent with cell death or atrophy in the cortex, we also saw decreases in neurite density, as well as increases in neurite orientation dispersion in participants with central vision loss. The patterns of these changes across cortex suggest that the representation of the center of the fovea may not be as plastic as just outside the fovea. Further, the pattern of data provided no strong evidence for use‐dependent increases in cortical thickness, changes in neurite density, or changes in orientation dispersion in healthy vision. However, our results do suggest possible influences of age of disease onset on all of these structural measures. Early‐onset MD participants showed more distinct differences in thickness, neurite density, and orientation dispersion compared with their controls than late‐onset participants did, and cortical thickness results suggest that in the early‐onset population, there may be competing processes contributing to cortical thickening (at the PRL) in the context of overall visual cortical thinning associated with low vision. Future studies should examine how individual differences in experiences relate to individual differences in cortical thickness and NODDI values. By incorporating complex information about individuals' visual experiences, we can move the field toward understanding what aspects of large‐scale brain structure change with experience.

## CONFLICT OF INTEREST STATEMENT

The authors declare that there is no conflict of interest regarding the publication of this article.

## Supporting information


**Figure S1:** The participant reported the right eye as the better eye, the left eye was unable to be tracked during the exam.

**Figure S2:** Raw thickness across region of interest (ROI), diagnosis, and onset groups. Cortical thickness is shown at the lesion projection zone (LPZ), cPRL, and cURL as a function of both onset and diagnosis. Error bars represent standard error of the mean. LPZ thicknesses showed no significant differences as a function of either diagnosis (*F*[1, 30] = 0.298, *p* = .589) or interaction of onset and diagnosis (*F*[1, 30] = 0.849, *p* = .364). Differences in thicknesses at the cortical projection of the preferred retinal locus (cPRL) and projection of unpreferred retinal locus (cURL) were tested as a function of ROI location, onset, and diagnosis, and showed a significant main effect of diagnosis (*F*[1, 30] = 20.86, *p* < .001) as well as a significant interaction of diagnosis and onset (*F*[1, 30] = 6.32, *p* = .018).
**Figure S3:** raw thickness across eccentricity regions of interest (ROIs). Mean normalized thickness for each MD and control group at each eccentricity ROI are shown. Error bars represent ±1 SEM. Values shown on the *x*‐axis correspond to the minimum eccentricity for that ROI. A three‐way ANOVA of cortical thickness as a function of diagnosis, onset, and ROI was performed. Main effects of diagnosis (*F*[1, 30] = 10.23, *p* = .003) as well as ROI (*F*[4.01, 120.26] = 16.37, *p* = 8.46 × 10^−11^) were significant. The main effect of ROI did not pass Mauchley's test of sphericity (*W* = 0.061, *p* = 1.4 × 10^−6^) and so the degrees of freedom were adjusted using Hyun–Feldt epsilon (*ε*_HF_ = 0.573). No two‐way or three‐way interactions were significant.

## Data Availability

Data will be made available as part of the Human Connectome Project Connectomes in Human Diseases online repository. Before full archiving and publication of that resource, please contact the authors for access to the data. All code used in these analyses are available at https://github.com/Visscher-Lab/Defenderfer_2022_HBM.
