# Supplementary material for: Cortical plasticity in central vision loss: Cortical thickness and neurite structure
Source: Hum Brain Mapp. 2023 May 17;44(10):4120–35. doi: 10.1002/hbm.26334 (PMC10258531; doi:10.1002/hbm.26334)
Supplement: Supplementary file 1 — Figure S1: The participant reported the right eye as the better eye, the left eye was unable to be tracked during the exam. [file HBM-44-4120-s002.pdf]

MDP004

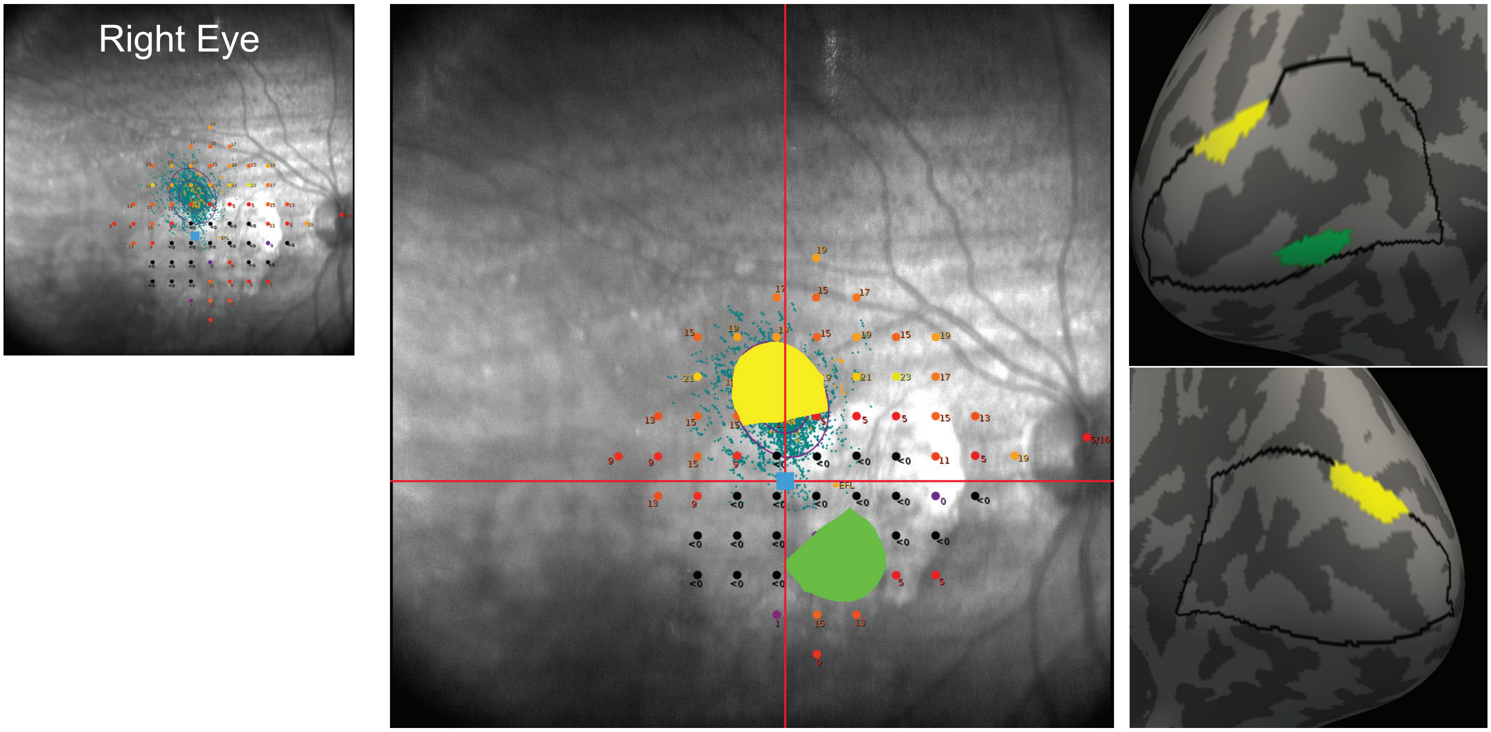

1. The participant reported the right eye as the better eye, the left eye was unable to be tracked during the exam.

MDP005

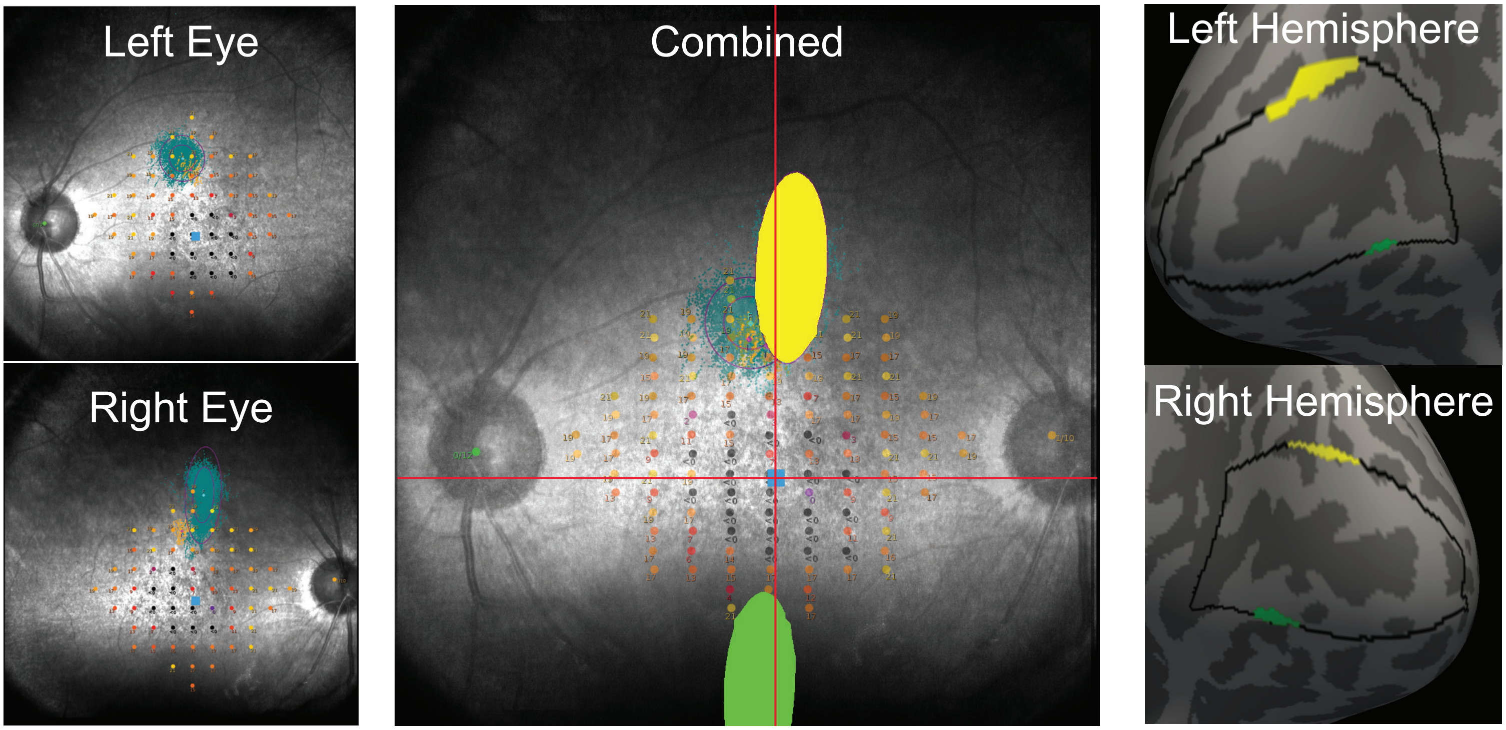
Left Eye
Combined
Left Hemisphere
Right Eye
Right Hemisphere

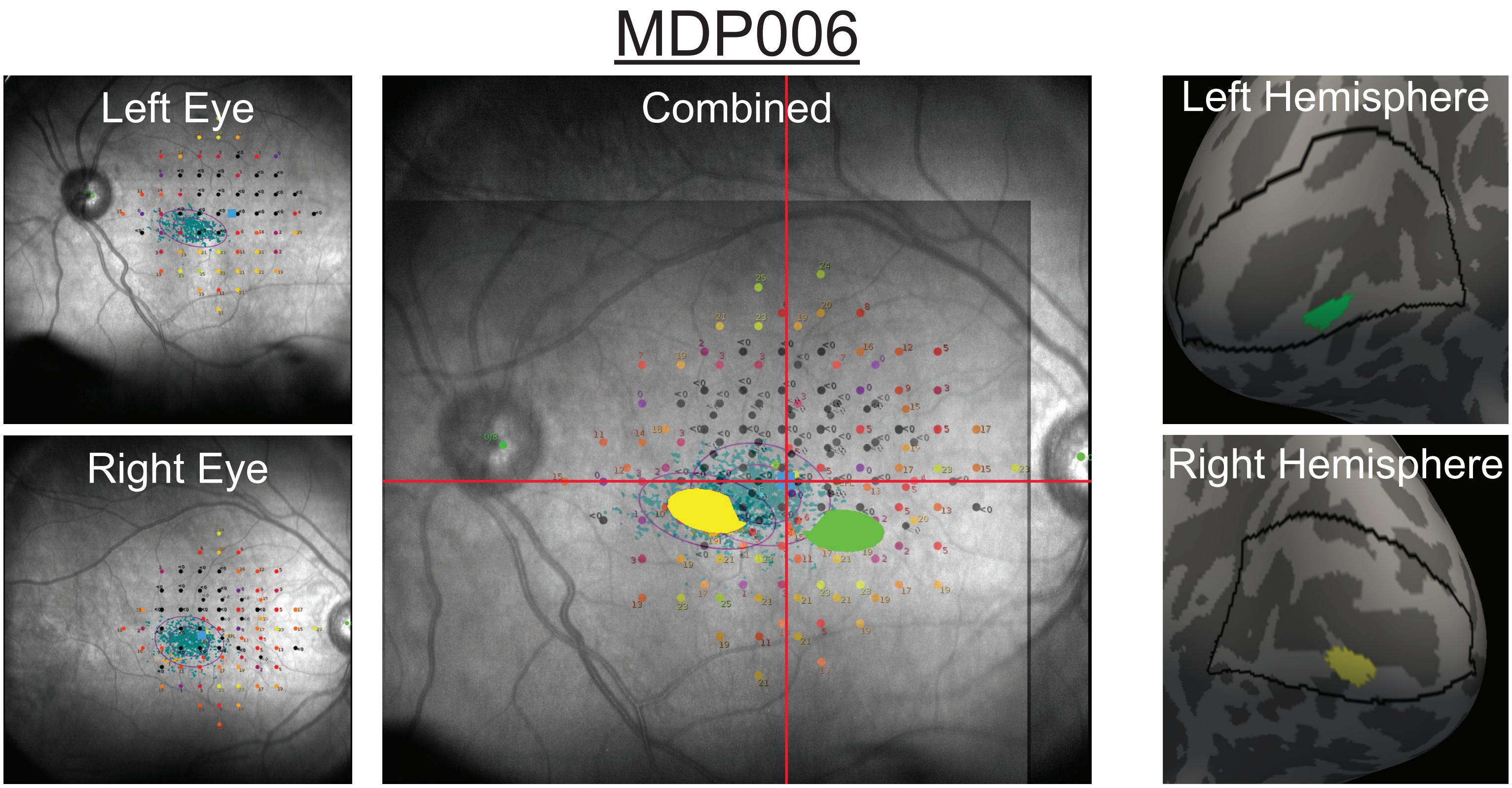
MDP006
Left Eye
Right Eye
Combined
Left Hemisphere
Right Hemisphere

MDP008

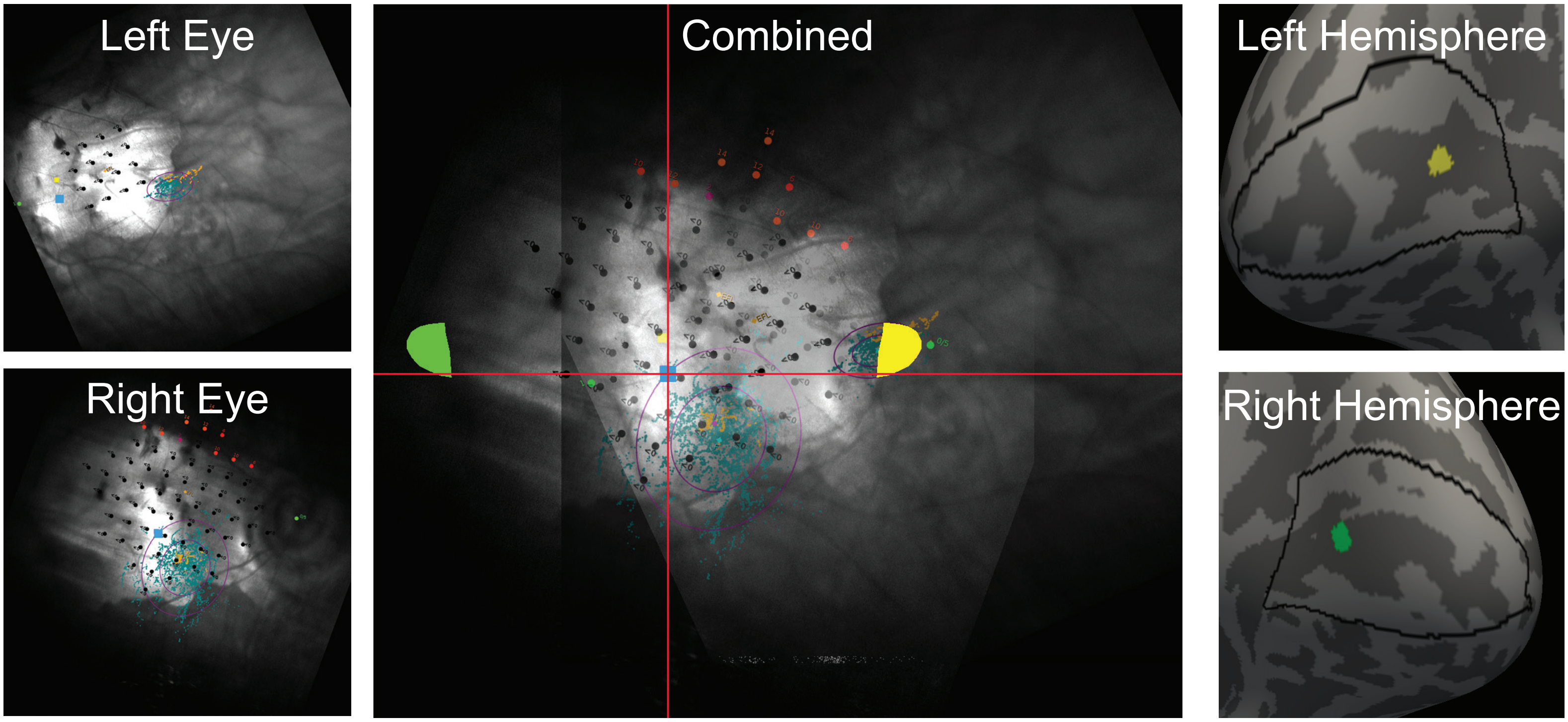

1. The participant reported the left eye as the better eye but was unable to finish the task with the larger stimulus grid. Visual sensitivity was unable to be measured at the PRL, so the ROI was limited to be the area outside of the scotoma visible using the MAIA

MDP014

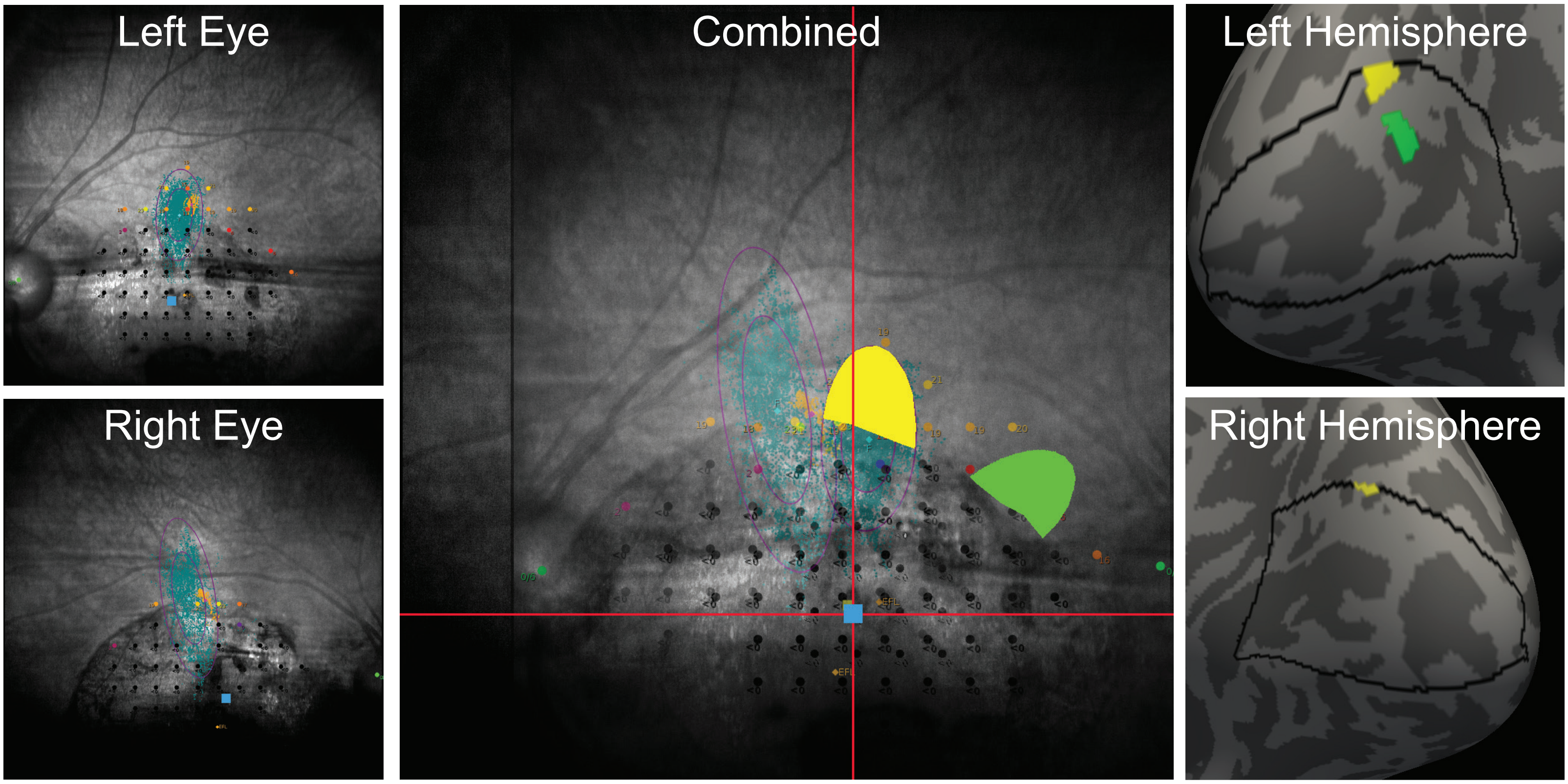
Left Eye
Combined
Left Hemisphere
Right Eye
Right Hemisphere

MDP016

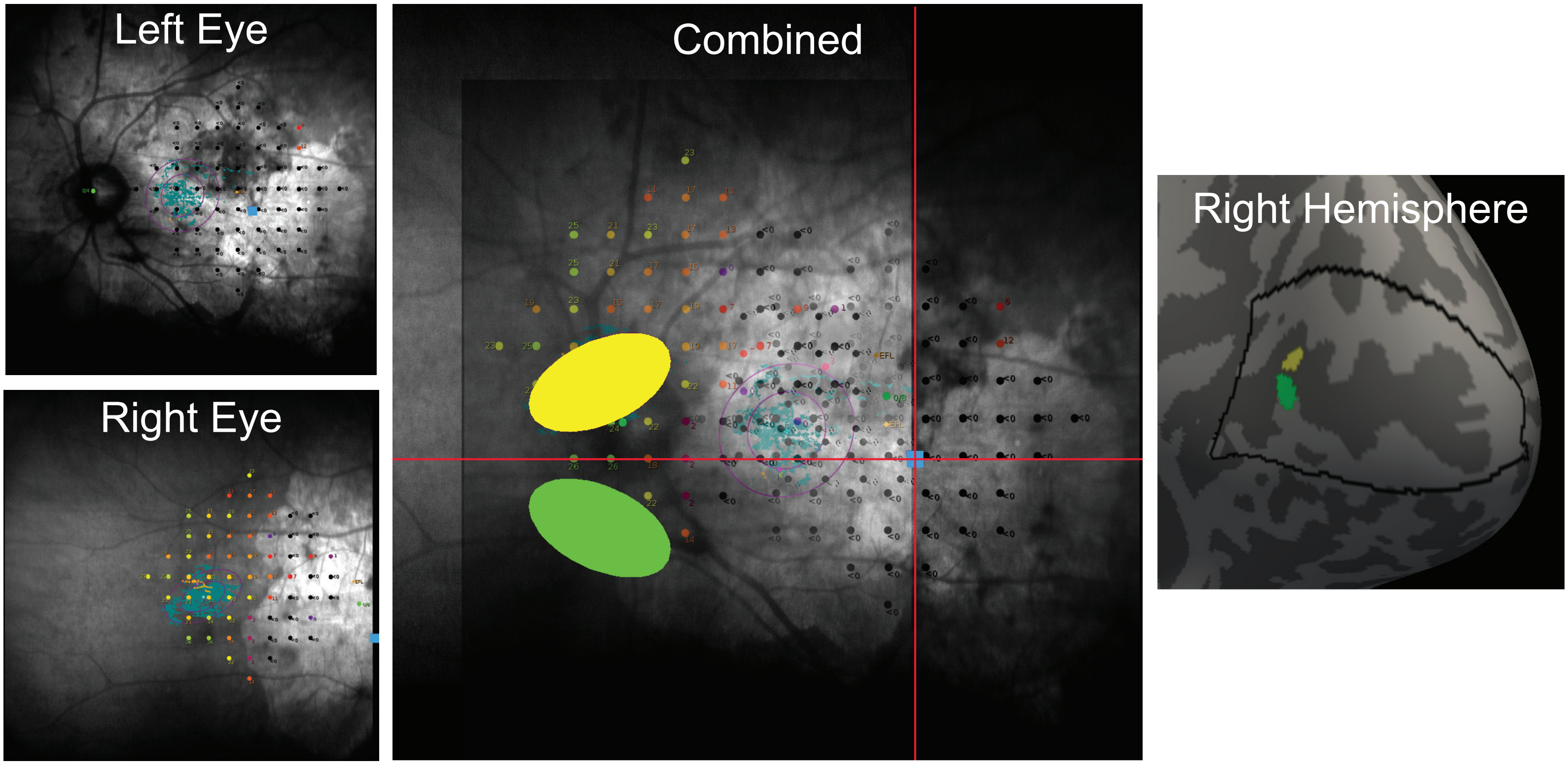

1. The participant reported the right eye as their better eye. The PRL for the right eye was located far enough in the periphery that the optic disc is not visible at all, and the fovea is not immediately apparent. Foveal location was estimated using the participant’s fundus photography where the optic disc was clearly visible. Lesion landmarks were used to estimate the foveal location in the MAIA image using the fundus photography as a guide

MDP021

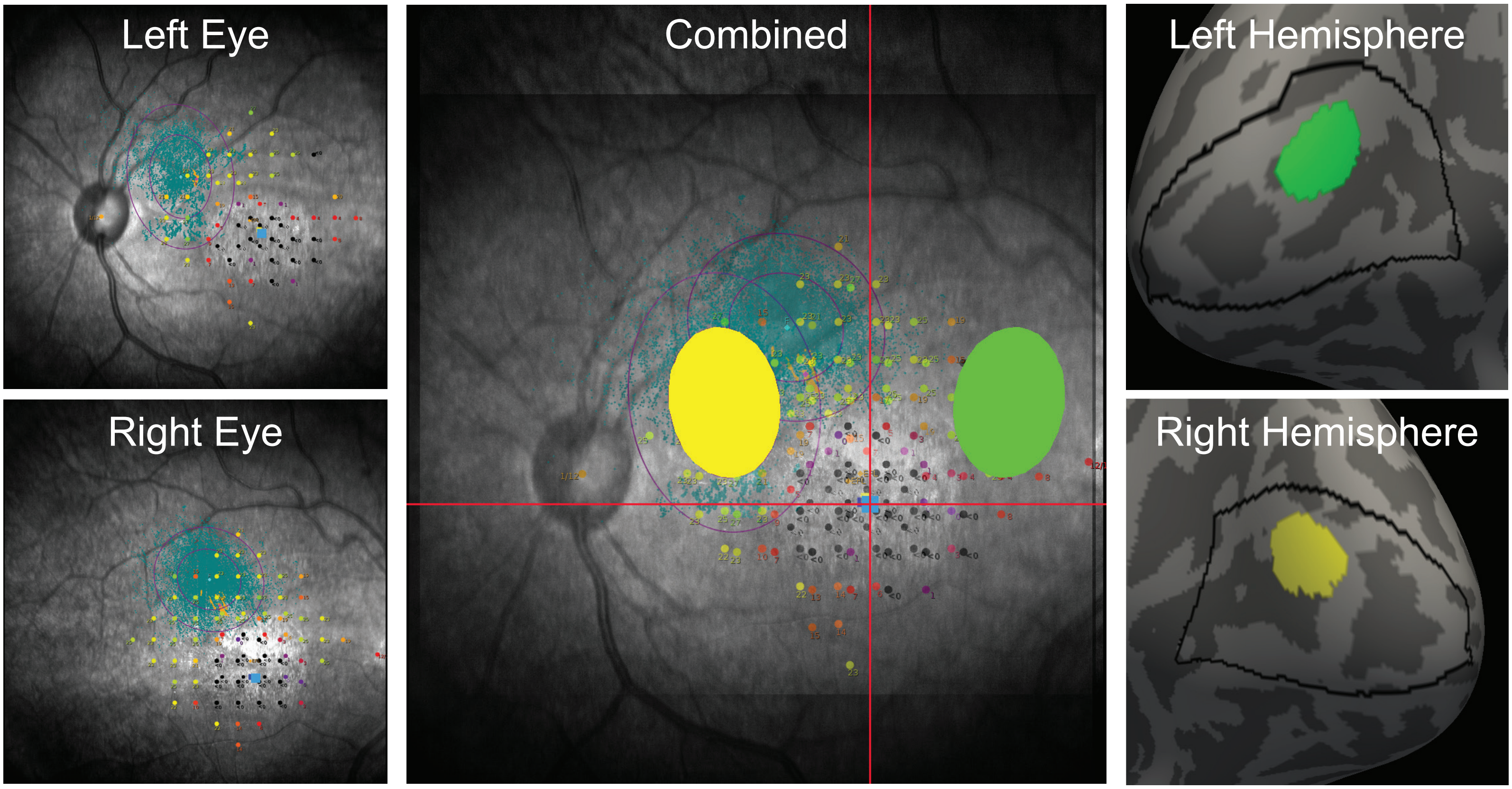

Left Eye
Combined
Left Hemisphere
Right Eye
Right Hemisphere

MDP022

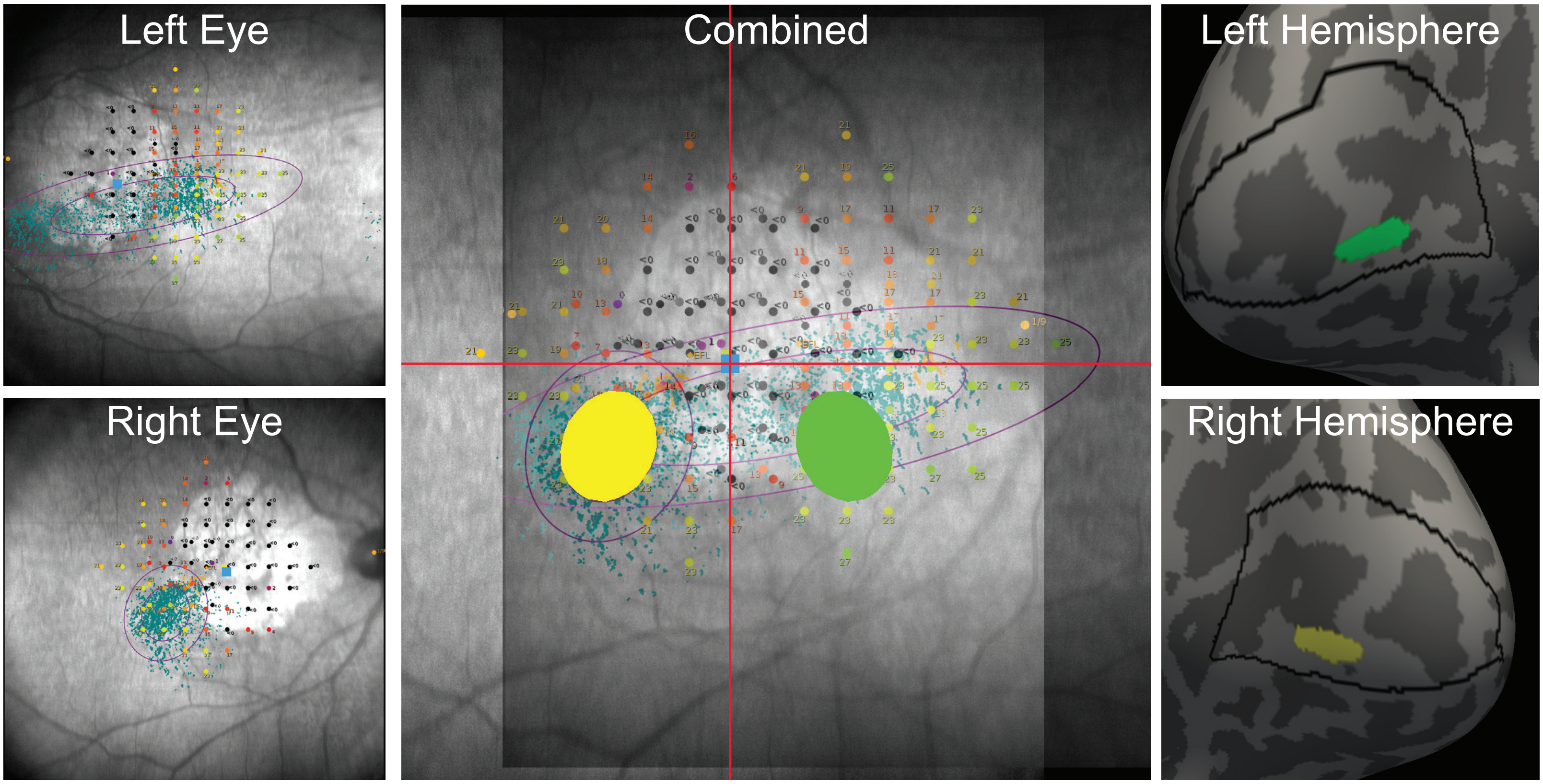

1. Left eye fovea was marked using the participant's fundus photography data where the optic disc is visible.

MDP023

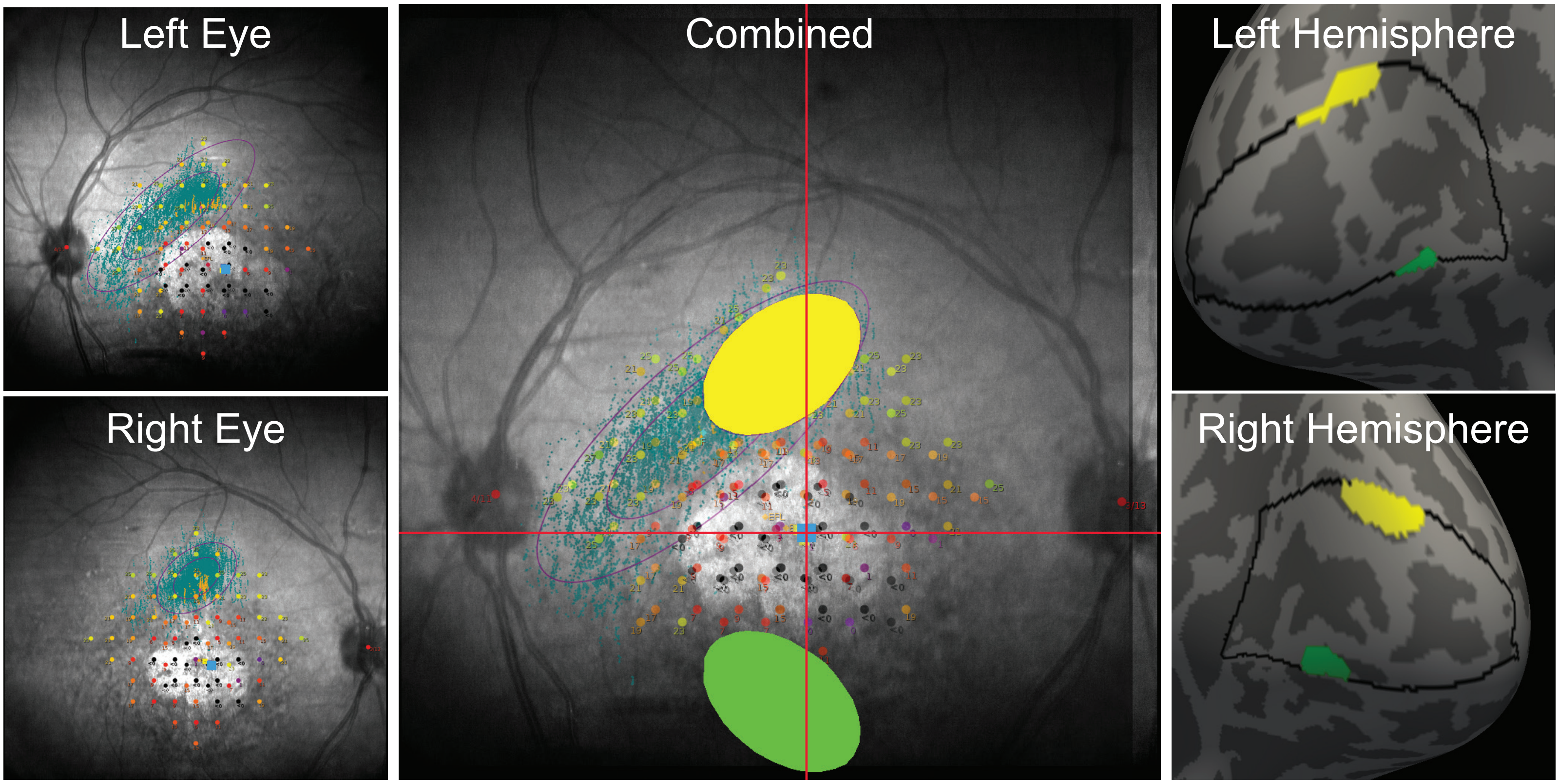
Left Eye
Right Eye
Combined
Left Hemisphere
Right Hemisphere

MDP027

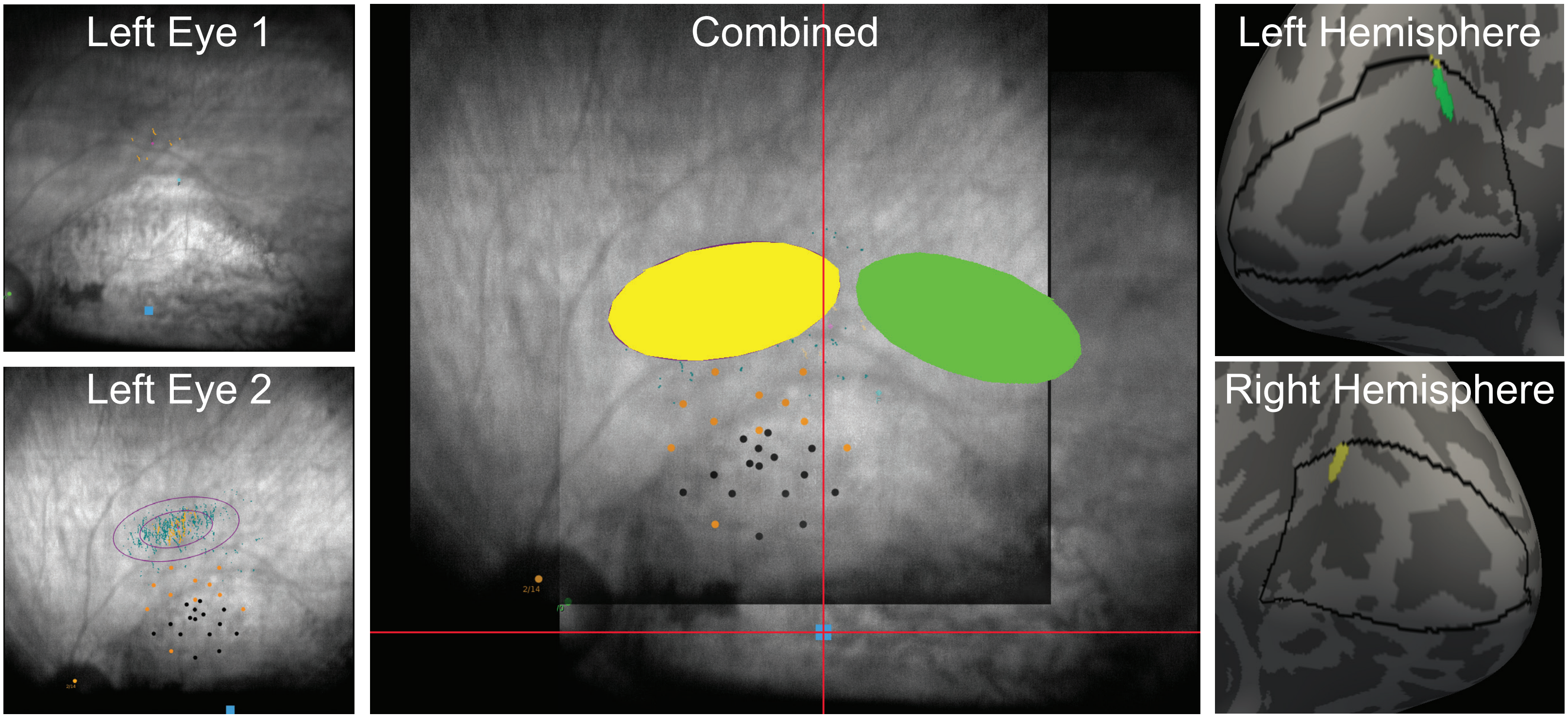

1. The participant had very poor fixation during the exam. The MAIA was unable to perform the full tasks due to poor eye tracking, so a shorter task was performed instead. This task was a binary exam asking if the participant was able to detect the stimulus at highest intensity. Orange dots indicate a positive response while black dots indicate no response.
2. No data from the right eye was able to be acquired.
3. Two images were acquired for the left eye. Left eye 1 was used to better localize the fovea since more of the optic disc is visible, although no microperimetry data was able to be acquired during it.

MDP043

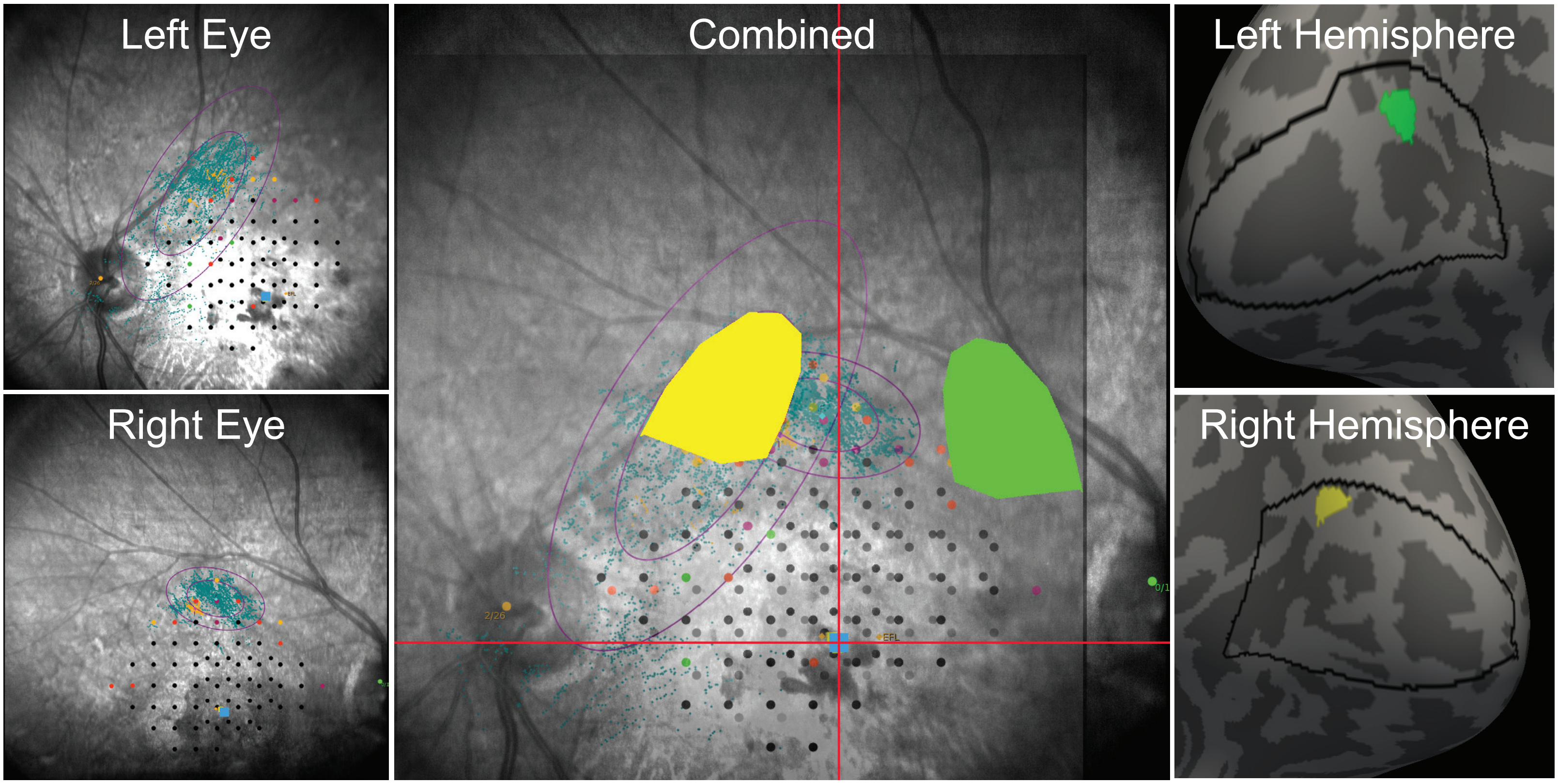

1. During the test, the participant lost visual contact with the fixation location for brief periods. Visual search caused the BCEA to be much greater than what seemed reasonable given that a large number of fixation points were within an area approximately half the size of the 63% BCEA. The PRL area (and therefore the URL area) was drawn to only include the location where most of the fixations occurred.

MDP047

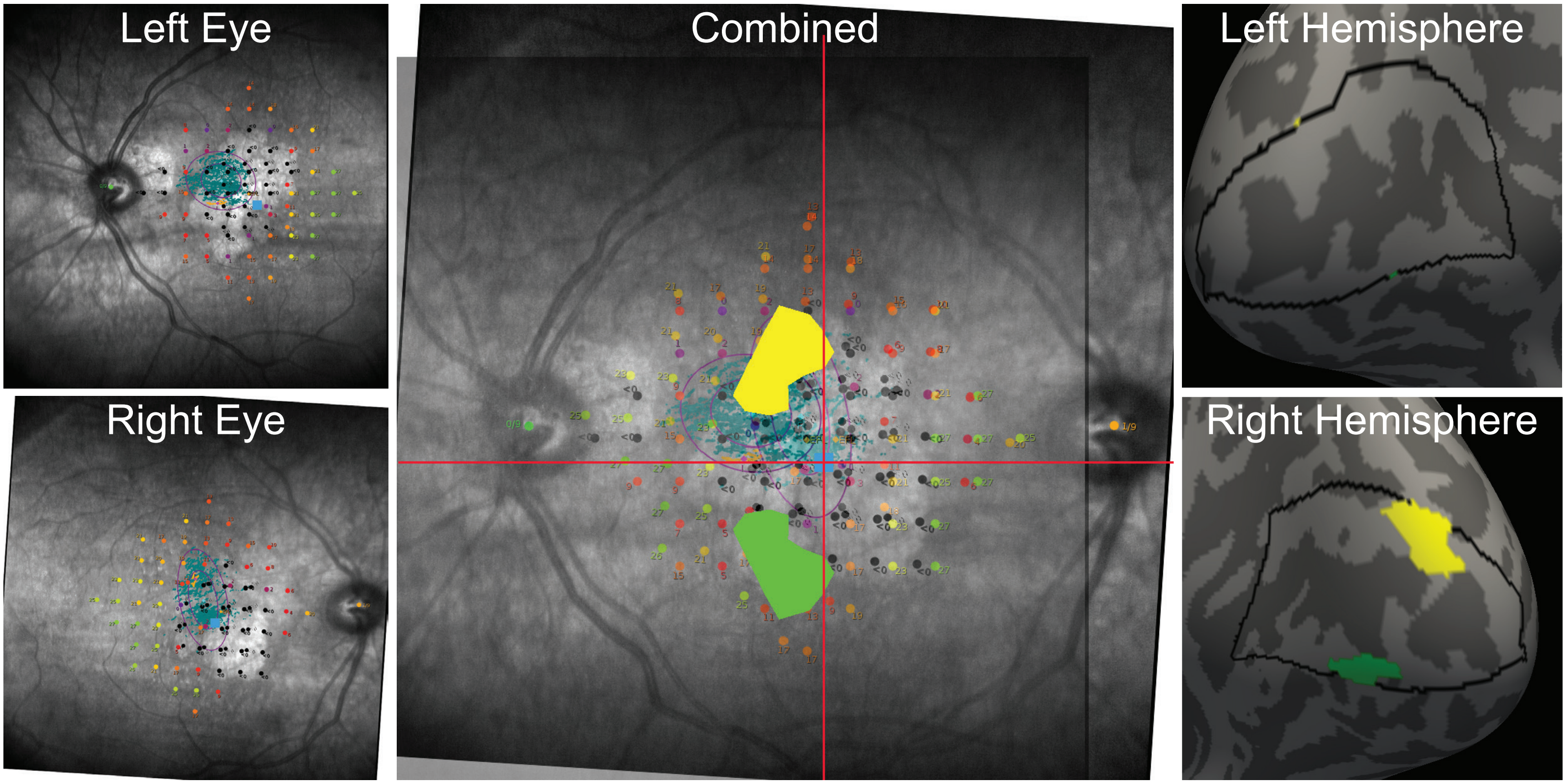

1. After examination, fixations in the participant’s better eye were determined to comprise two separate fixation locations. The PRL was defined as the area covering the more eccentric fixations outside the lesioned area in the better eye.

MDP050

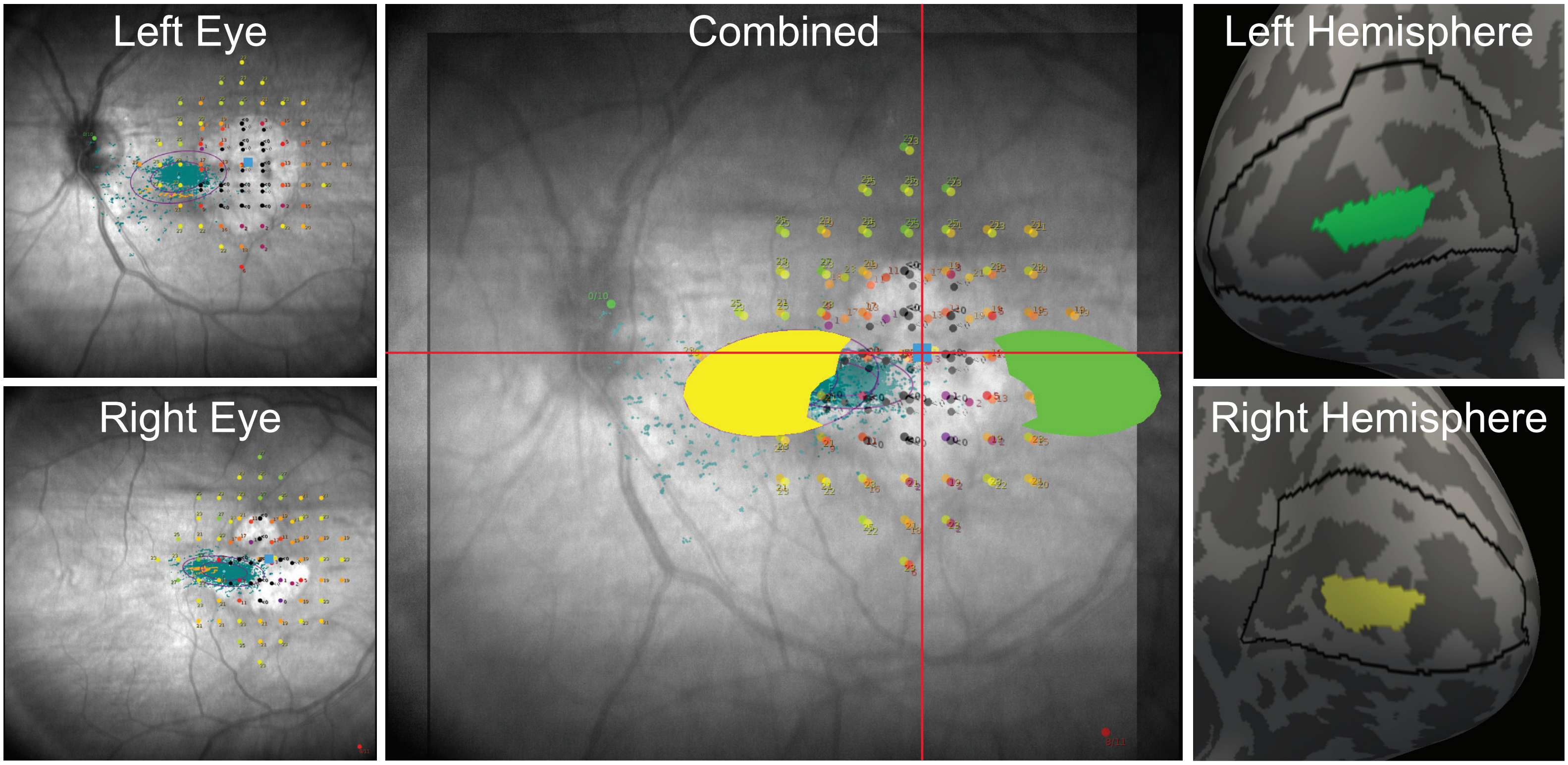
Left Eye
Right Eye
Combined
Left Hemisphere
Right Hemisphere

MDP065

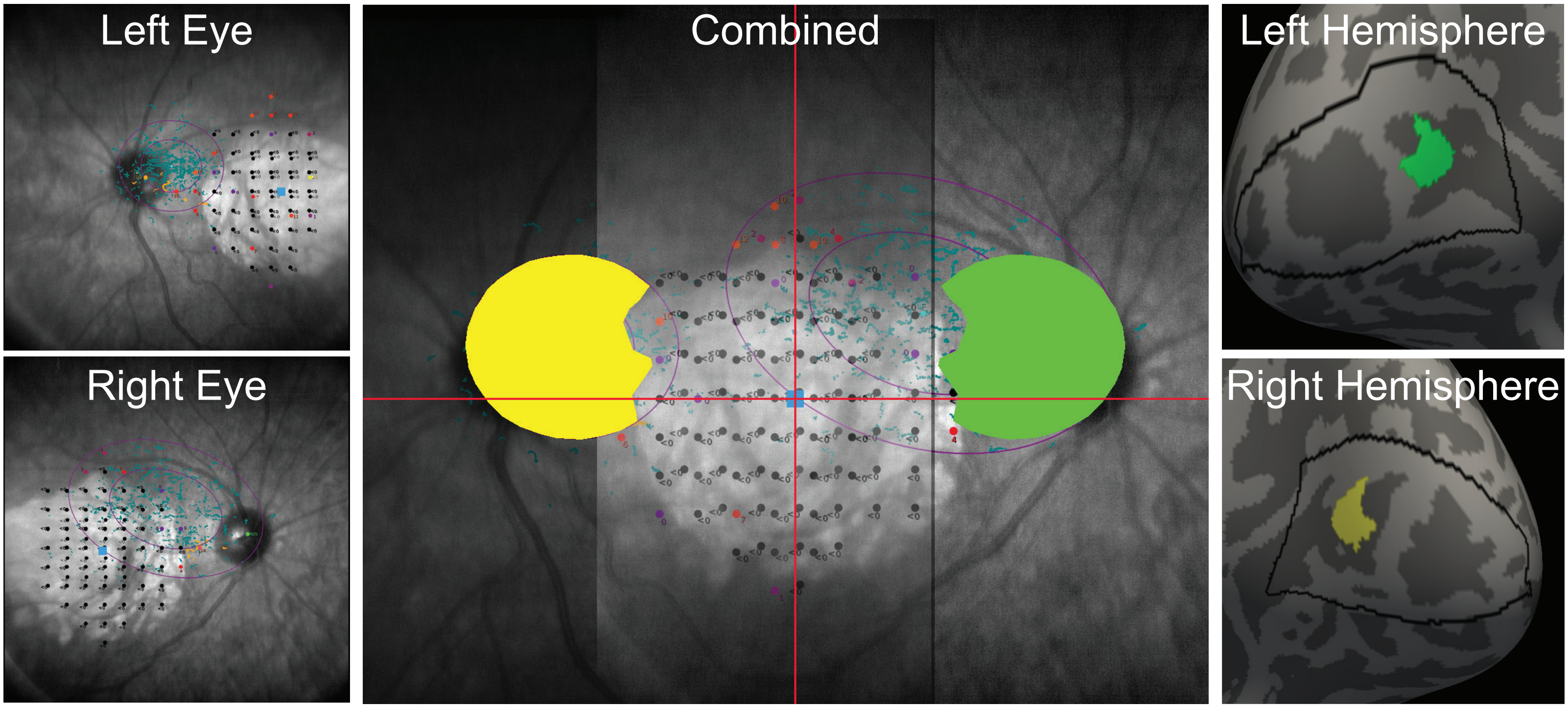
Left Eye
Combined
Left Hemisphere
Right Eye
Right Hemisphere

MDP117

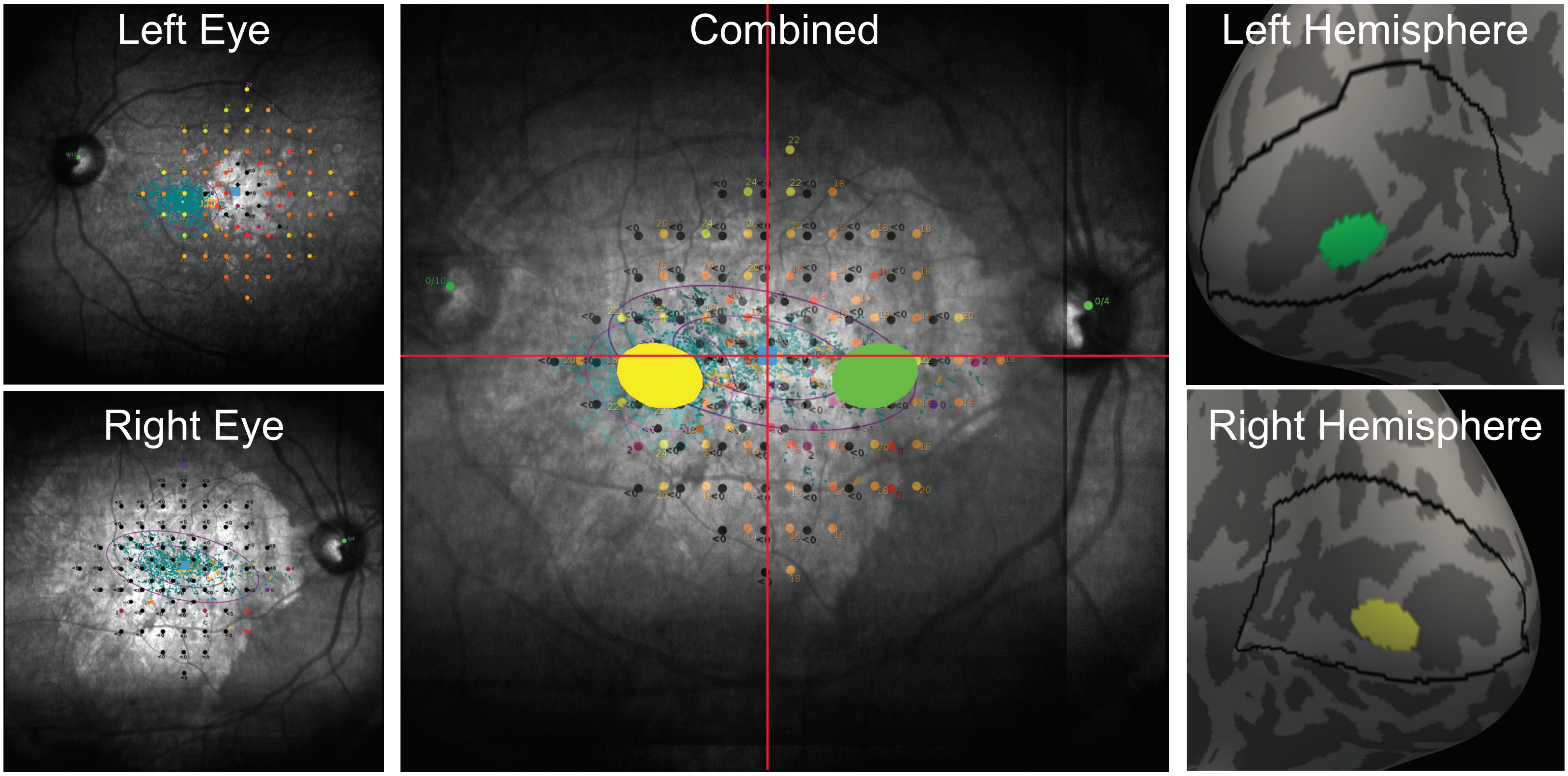
Left Eye
Combined
Left Hemisphere
Right Eye
Right Hemisphere

MDP122

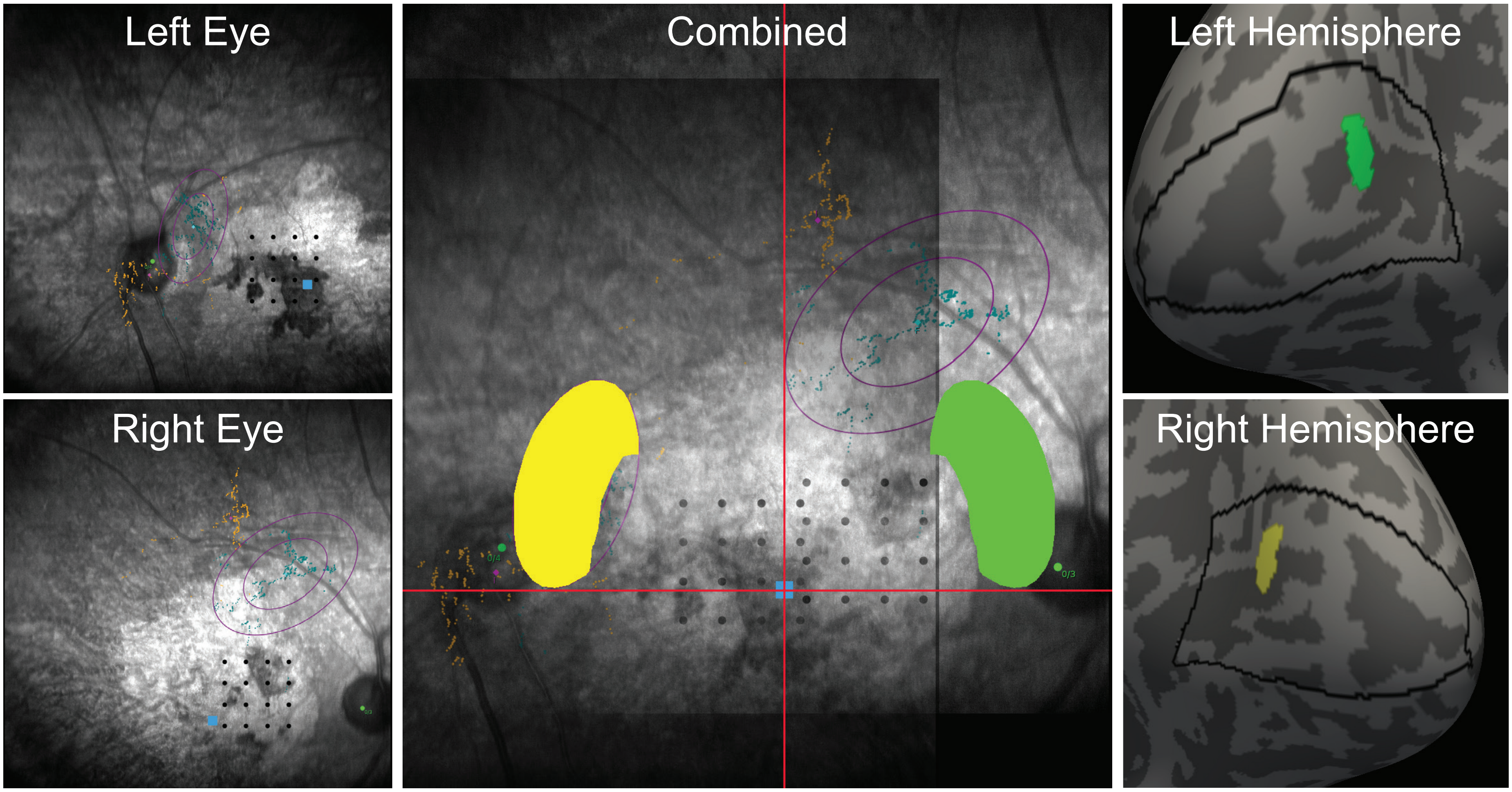

1. Participant had trouble performing the MAIA task with the full grid, so a smaller grid was used in each eye to test for retained central vision and have some measure of PRL location. The PRL was drawn to exclude the areas where the anatomical scotoma from both eyes overlapped.

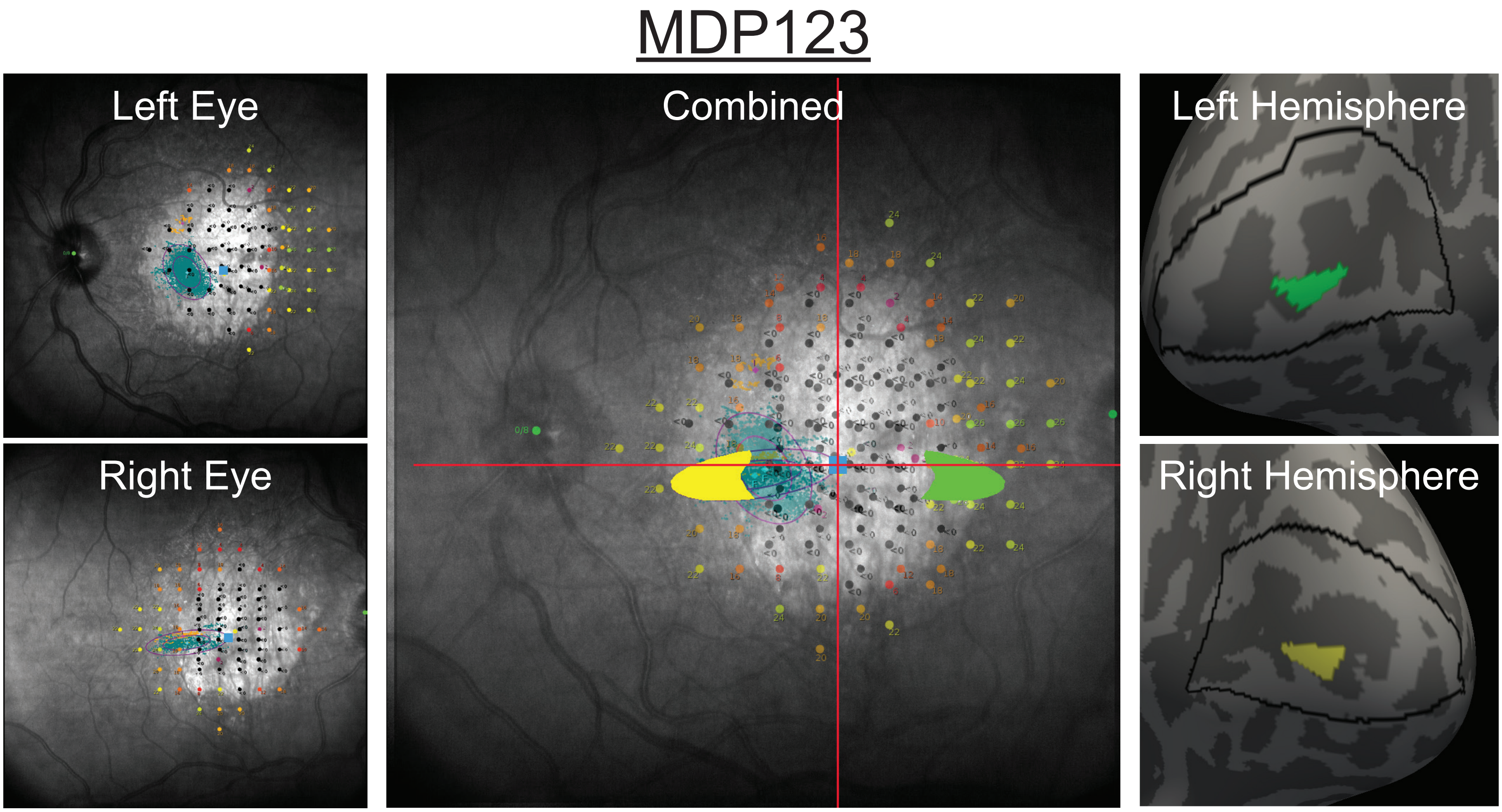

MDP123
Left Eye
Right Eye
Combined
Left Hemisphere
Right Hemisphere

MDP126

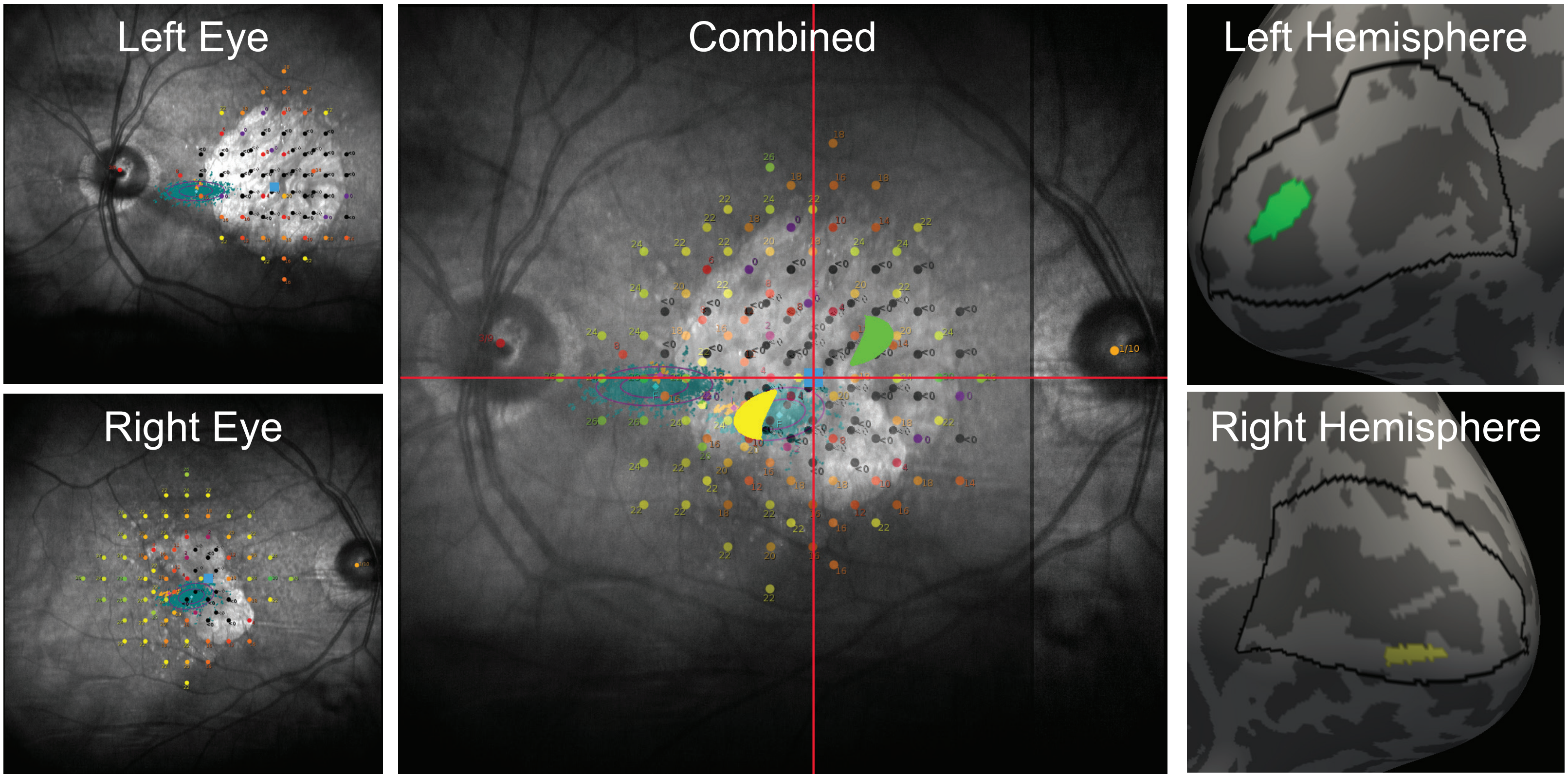

Left Eye
Combined
Left Hemisphere
Right Eye
Right Hemisphere

MDP142

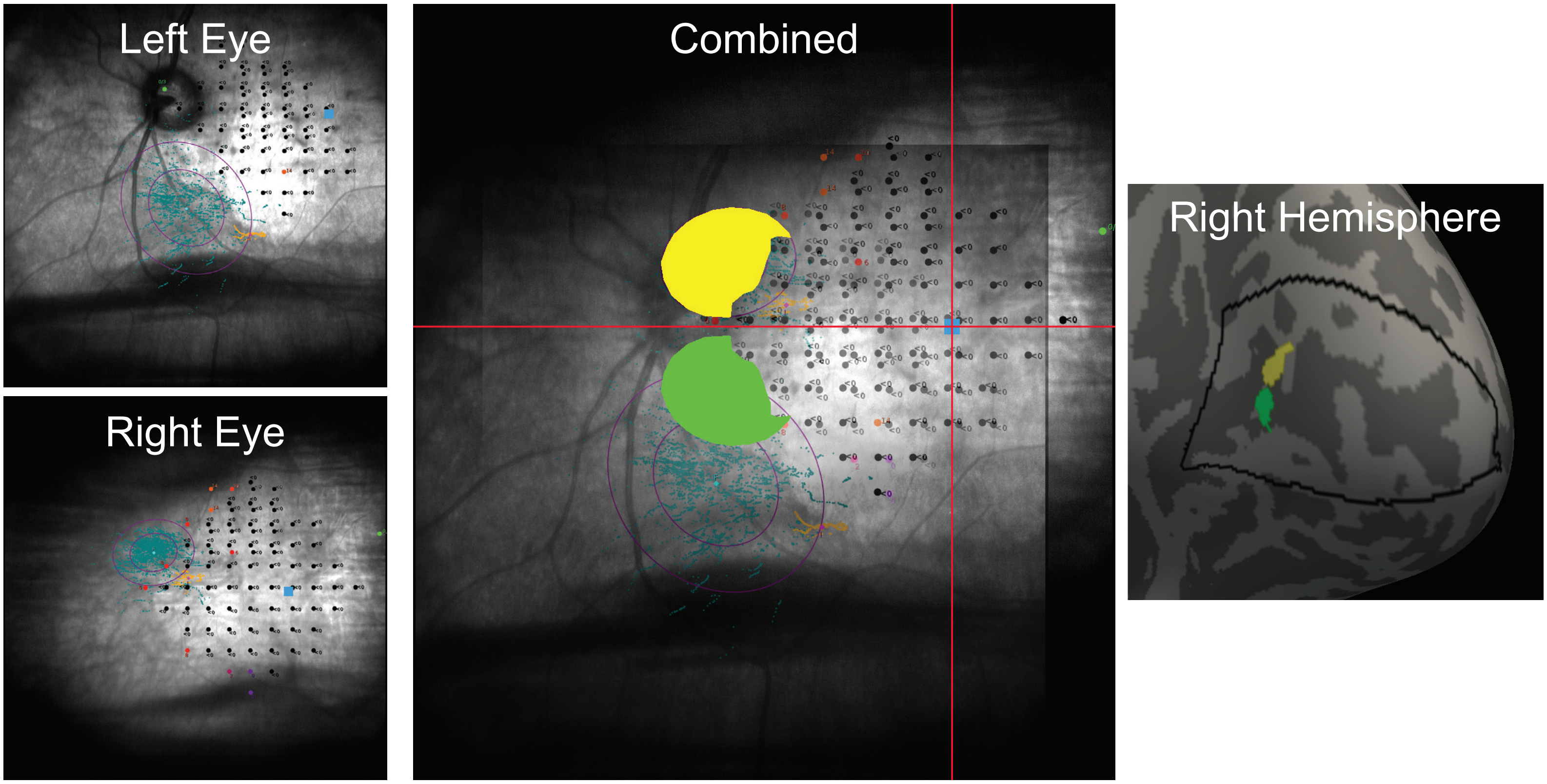

MDP174

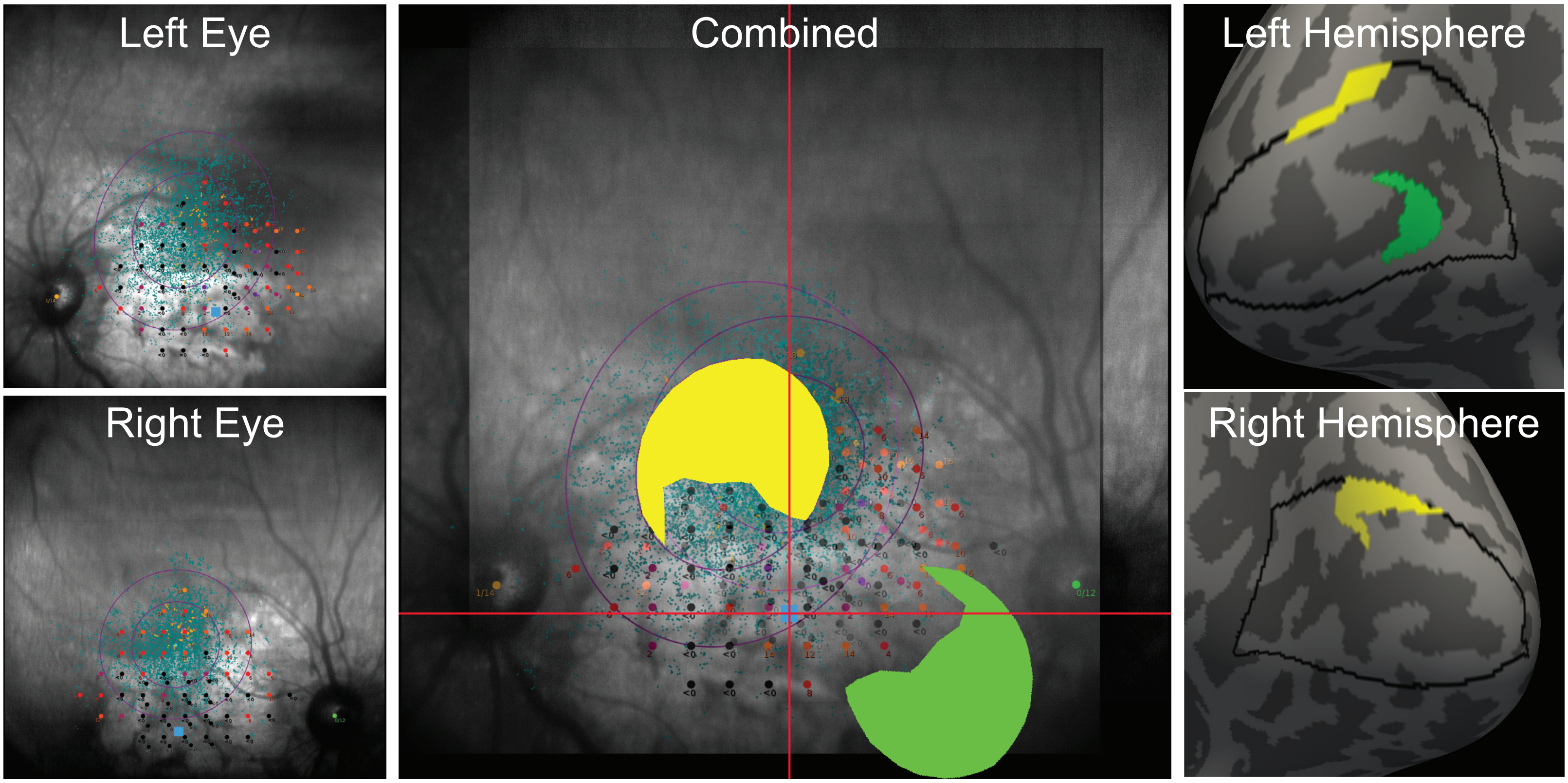
Left Eye
Right Eye
Combined
Left Hemisphere
Right Hemisphere
