# Supplementary material for: Cortical plasticity in central vision loss: Cortical thickness and neurite structure
Source: Hum Brain Mapp. 2023 May 17;44(10):4120–35. doi: 10.1002/hbm.26334 (PMC10258531; doi:10.1002/hbm.26334)
Supplement: Supplementary file 2 — Figure S2: Raw thickness across region of interest (ROI), diagnosis, and onset groups. Cortical thickness is shown at the lesion projection zone (LPZ), cPRL, and cURL as a function of both onset and diagnosis. Error bars represent standard error of the mean. LPZ thicknesses showed no significant differences as a function of either diagnosis (F[1, 30] = 0.298, p = .589) or interaction of onset and diagnosis (F[1, 30] = 0.849, p = .364). Differences in thicknesses at the cortical projection of the preferred retinal locus (cPRL) and projection of unpreferred retinal locus (cURL) were tested as a function of ROI location, onset, and diagnosis, and showed a significant main effect of diagnosis (F[1, 30] = 20.86, p < .001) as well as a significant interaction of diagnosis and onset (F[1, 30] = 6.32, p = .018). Figure S3: raw thickness across eccentricity regions of interest (ROIs). Mean normalized thickness for each MD and control group at each eccentricity ROI are shown. Error bars represent ±1 SEM. Values shown on the x‐axis correspond to the minimum eccentricity for that ROI. A three‐way ANOVA of cortical thickness as a function of diagnosis, onset, and ROI was performed. Main effects of diagnosis (F[1, 30] = 10.23, p = .003) as well as ROI (F[4.01, 120.26] = 16.37, p = 8.46 × 10^−11^) were significant. The main effect of ROI did not pass Mauchley's test of sphericity (W = 0.061, p = 1.4 × 10^−6^) and so the degrees of freedom were adjusted using Hyun–Feldt epsilon (ε HF = 0.573). No two‐way or three‐way interactions were significant. [file HBM-44-4120-s001.pdf]

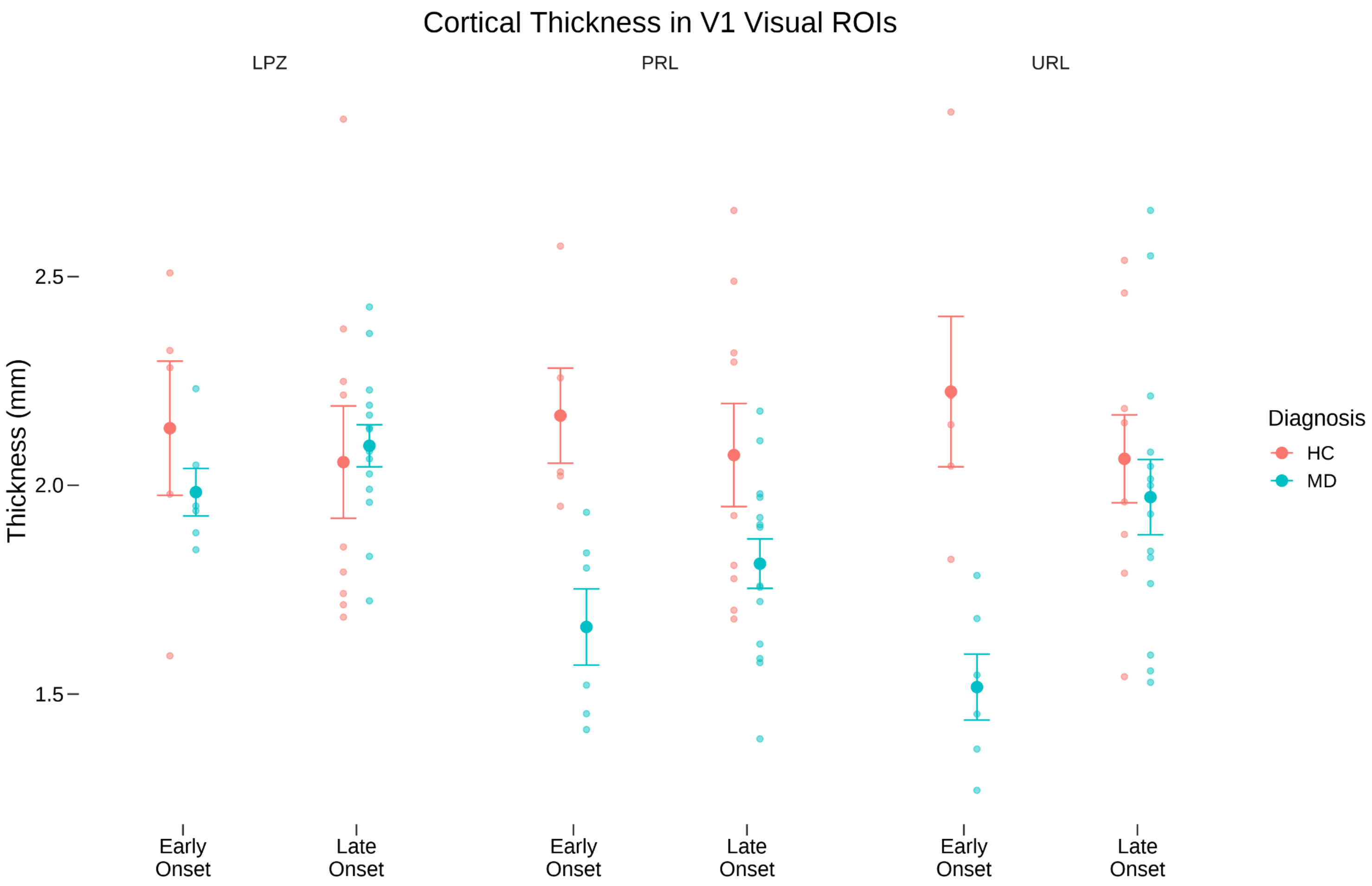

Figure S2. Raw Thickness Across ROI, Diagnosis, and Onset Groups. Cortical thickness is shown at the LPZ, cPRL, and cURL as a function of both onset and diagnosis. Error bars represent standard error of the mean. LPZ thicknesses showed no significant differences as a function of either diagnosis ($F(1,30) = 0.298$, $p = 0.589$) or interaction of onset and diagnosis ($F(1,30) = 0.849$, $p = 0.364$). Differences in thicknesses at the cPRL and cURL were tested as a function of ROI location, onset, and diagnosis, and showed a significant main effect of diagnosis ($F(1,30) = 20.86$, $p < 0.001$) as well as a significant interaction of diagnosis and onset ($F(1,30) = 6.32$, $p = 0.018$).

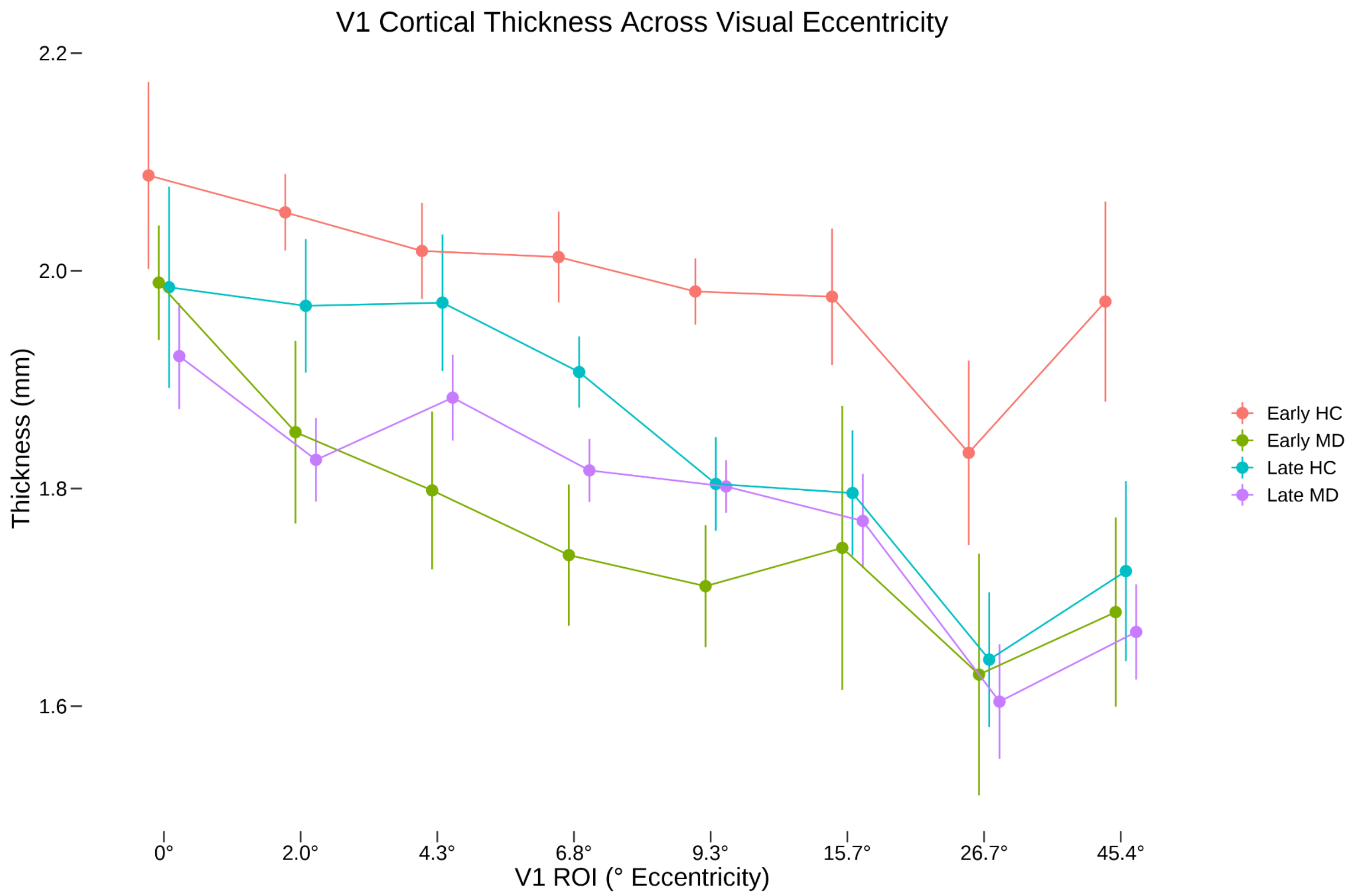

Figure S3. Raw Thickness Across Eccentricity ROIs. Mean normalized thickness for each MD and control group at each eccentricity ROI are shown. Error bars represent ±1 SEM. Values shown on the X-axis correspond to the minimum eccentricity for that ROI. A three-way ANOVA of cortical thickness as a function of diagnosis, onset, and ROI was performed. Main effects of diagnosis ($F(1,30) = 10.23$, $p = 0.003$) as well as ROI ($F(4.01,120.26) = 16.37$, $p = 8.46*10^{-11}$) were significant. The main effect of ROI did not pass Mauchley's test of sphericity ($W = 0.061$, $p = 1.4*10^{-6}$) and so the degrees of freedom were adjusted using Hyun-Feldt epsilon ($\varepsilon_{HF} = 0.573$). No two-way nor three-way interactions were significant.
